# Critical Neurotransmitters in the Neuroimmune Network

**DOI:** 10.3389/fimmu.2020.01869

**Published:** 2020-08-21

**Authors:** Thomas Wesley Hodo, Maria Teresa Prudente de Aquino, Akiko Shimamoto, Anil Shanker

**Affiliations:** ^1^Department of Biochemistry, Cancer Biology, Neuroscience and Pharmacology, Meharry Medical College School of Medicine, Nashville, TN, United States; ^2^Department of Microbiology and Immunology, Meharry Medical College School of Medicine, Nashville, TN, United States; ^3^School of Graduate Studies and Research, Meharry Medical College, Nashville, TN, United States; ^4^Host-Tumor Interactions Research Program, Vanderbilt-Ingram Cancer Center, Vanderbilt University Medical Center, Nashville, TN, United States; ^5^Vanderbilt Center for Immunobiology, Vanderbilt University Medical Center, Nashville, TN, United States; ^6^Vanderbilt Institute for Infection, Immunology and Inflammation, Vanderbilt University Medical Center, Nashville, TN, United States

**Keywords:** T cell neuroimmunology, dopamine, glutamate, serotonin, substance P, cancer, neurodegenerative disorders, immunotherapy

## Abstract

Immune cells rely on cell-cell communication to specify and fine-tune their responses. They express an extensive network of cell communication modes, including a vast repertoire of cell surface and transmembrane receptors and ligands, membrane vesicles, junctions, ligand and voltage-gated ion channels, and transporters. During a crosstalk between the nervous system and the immune system these modes of cellular communication and the downstream signal transduction events are influenced by neurotransmitters present in the local tissue environments in an autocrine or paracrine fashion. Neurotransmitters thus influence innate and adaptive immune responses. In addition, immune cells send signals to the brain through cytokines, and are present in the brain to influence neural responses. Altered communication between the nervous and immune systems is emerging as a common feature in neurodegenerative and immunopathological diseases. Here, we present the mechanistic frameworks of immunostimulatory and immunosuppressive effects critical neurotransmitters — dopamine (3,4-dihydroxyphenethylamine), serotonin (5-hydroxytryptamine), substance P (trifluoroacetate salt powder), and L-glutamate — exert on lymphocytes and non-lymphoid immune cells. Furthermore, we discuss the possible roles neurotransmitter-driven neuroimmune networks play in the pathogenesis of neurodegenerative disorders, autoimmune diseases, cancer, and outline potential clinical implications of balancing neuroimmune crosstalk by therapeutic modulation.

## Introduction

The nervous and immune systems present the body with two main interfaces to perceive, integrate, and respond to environmental insults or internal injuries. Both systems adapt to ever-changing conditions to mount their responses. While propounding his network theory of the immune system, Niels Jerne had highlighted functional similarities in the recognition mechanisms and memory formation capabilities of the nervous and immune systems (1). Epithelial cells and immune cells stand at the frontline defense against the tissue insults arising from trauma, injury, or infection. Recent advances in neuroscience have added neurons to the frontline arsenal with an intricate bidirectional communication between the nervous and immune systems. Altered communication between the two systems is emerging as a common feature in neurodegenerative and immunopathological diseases.

Experiments conducted by Medawar et al. in the late 1940s introduced the notion that the brain parenchyma is an immunoprivileged site on the basis of the observations that skin allografts implanted into the brain parenchyma elicited a delayed graft rejection (2). This was attributed to a lack of lymphatics and antigen presentation in the brain. Lately, it has been found that the blood-meningeal barrier is more permissive than the blood-brain barrier to immune cells. This allows immune cells to circulate within the meninges to carry out surveillance in the central nervous system (CNS) under homeostatic conditions and capture antigens draining from the brain parenchyma or cerebral spinal fluid via glymphatics into the cervical lymph nodes (LN) (3–5). Recently, using single-cell high-dimensional cytometry all subsets of immune cells were shown to be present in the brain (6–8). Inflammatory pathways triggered by cytokines produced by immune cells in the CNS in conjunction with CNS-infiltrating lymphocytes and “inflammaging” lie at the heart of neurodegenerative diseases (9, 10). There is also evidence of the modulation of anti-bacterial (11) and anti-tumor (12) immune responses by the brain's reward system. It is now known that highly invasive bacterial pathogens, such as *Streptococcus pyogenes* promote their survival by hijacking pain and neuronal regulation of the immune response (13). Thus, balancing neuroimmune crosstalk in diseased individuals may offer a novel strategy for therapeutic development to treat various pathologies.

Anatomical proximity and molecular mechanisms of communication, including receptors and signaling messengers, shared by the nervous and immune systems facilitate their crosstalk. Millions of years of evolutionary pressure has shaped the co-evolution of mammalian neuroimmune networks to maintain cellular and physiological homeostasis in the context of changes happening in the body's external and internal (blood pressure, pH, or temperature) environments. Despite an exciting progress over the last decades in understanding the partnership of neuroscience and immunology, precise mechanisms of neuroimmune crosstalk remain enigmatic (14, 15). Here, we review the known mechanistic frameworks of neuroimmune networks with respect to critical neurotransmitters.

## Neuroimmune Crosstalk During Immune Ontogeny, Cellular Programming and Function

Neuroimmune crosstalk can be traced to the early steps of immune system ontogenesis. Hematopoietic stem cell (HSC) niche-forming mesenchymal stem cells (MSC) in perinatal bone marrow arise from the neural crest. These niche-forming MSCs share a common origin with peripheral sympathetic neurons and Schwann cells, an ontogenic relationship that underscores the sympathetic regulation of adult HSC activity (16). Schwann cells ensheath bone marrow nerves and control HSC quiescence through activation of latent transforming growth factor β (TGFβ) (17) or HSC mobilization through neuron-derived catecholamines or norepinephrine (18, 19). CNS-resident immune cells include microglia, perivascular, meningeal and choroid plexus macrophages as well as astrocytes. Microglia arise from the embryonic yolk sac erythromyeloid precursors (EMP), which are also thought to give rise to macrophages in other tissues of the body (20). EMPs penetrate the CNS early during development (21).

Neuroimmune interactions guide cellular programming in tissues. This is evident, for example, in the framework of enteric nervous system and intestinal macrophages. In the gut, lamina propria macrophages (LpM) exhibit an inflammatory while muscularis macrophages (MM) anti-inflammatory tissue-protective phenotypes. Upon luminal bacterial infection, MMs enhance tissue-protective programs (e.g., increased expression of *Arg1* and *Chi3I* genes), accumulate near sympathetic nerve fibers and present high levels of β2 adrenergic receptors (β2AR) for norepinephrine signaling (22). Such a cellular network between enteric neurons and macrophages affords intra-tissue adaptation to protect from distal threats.

Vasoconstriction-stimulatory adrenergic nerve innervation of the peripheral secondary lymphoid organs, such as spleen, observed in mammals signifies a functional layer of CNS influence on immune system (23, 24). Indeed, catecholamines produced by the adrenal medulla and the postganglionic fibers of the sympathetic nervous system (SNS) affect immune cell activation, proliferation, and apoptosis. It is postulated that neuroimmune functional crosstalk evolved, for instance, to eliminate parasites that cannot be taken care of by cell-mediated immunity (25). The sensation of itch as a parasite invades the skin leads to a reflex action of scratching; worms in the gut initiate peristalsis; and parasites in the lung lead to cough and enhanced mucus production. In these situations, T helper-2 (T_H2_)-stimuli activate sensory neurons in tissues, and parasites are eliminated by a joint neuroimmune defense: a release of histamine, T_H2_ response in the abundance of interleukin-4 (IL-4) secretion (which sensitize nociceptors), and production of mucus (due to neuroimmune effort) and IgE antibodies.

Immune cells also provide signals that impact the nervous system. Besides sending signals to the brain through cytokines, peripheral immune cells are present in the brain to influence neural responses. For example, IFNγ-producing T cells are predominant in the subventricular zone of aged brains, where they suppress proliferation of neural stem cells (26). In a stroke, regulatory T (T_reg_) cells accumulate in the brain to accelerate neurological recovery (27). Moreover, during hypothermia macrophages become an alternative source of catecholamines such as noradrenaline to sustain thermogenesis (28, 29) through activation of the sympathetic nervous system pathway in warm-blooded (homeotherm) animals. Macrophages in adipose tissues of mice exposed to cold temperatures upon activation through IL-4/IL-13-dependent mechanisms produce noradrenaline, which stimulates β3 adrenergic receptors and activates thermogenic genes (29). Therefore, neurotransmitters through their defined signals can play a variety of roles in maintaining the body homeostasis.

## CNS Links to Innate Inflammatory Responses and Adaptive Immune Cells

CNS-resident immune cells including microglial, perivascular, meningeal, and choroid plexus macrophages as well as astrocytes participate in an innate inflammatory response upon engagement of their Toll-like receptors (TLR) with highly conserved motifs of danger-associated molecular patterns (DAMP) or pathogen-associated molecular patterns (PAMP) on stressed or infected tissue. This response can damage neurons and other brain cells through chronic production of pro-inflammatory cytokines and other mediators. A well-established feedback circuit exists between inflammatory cytokines and the hypothalamic-pituitary-adrenal (HPA) axis, a major brain-associated regulatory mechanism underlying stress responses (30–32). The HPA axis also plays a critical role in controlling the physiology and function of thymus, the T cell production center and a sensor of immunological homeostasis in the body (33–35). The studies conducted by Seyle in 1936 showed that various physiologic or pathologic stress stimuli led to adrenal enlargement and involution of thymus (36). Subsequent studies in mice and humans confirmed that the activation of HPA axis caused thymic involution (37–39). Studies have also shown in murine models that progressive ascitic growth of lymphomas producing an immunosuppressive inflammatory environment causes thymic atrophy (40, 41), which could be reversed with the administration of thymic hormones (42, 43). These observations point to a role of HPA-thymus axis in the control of immunological homeostasis.

**Figure 1:**
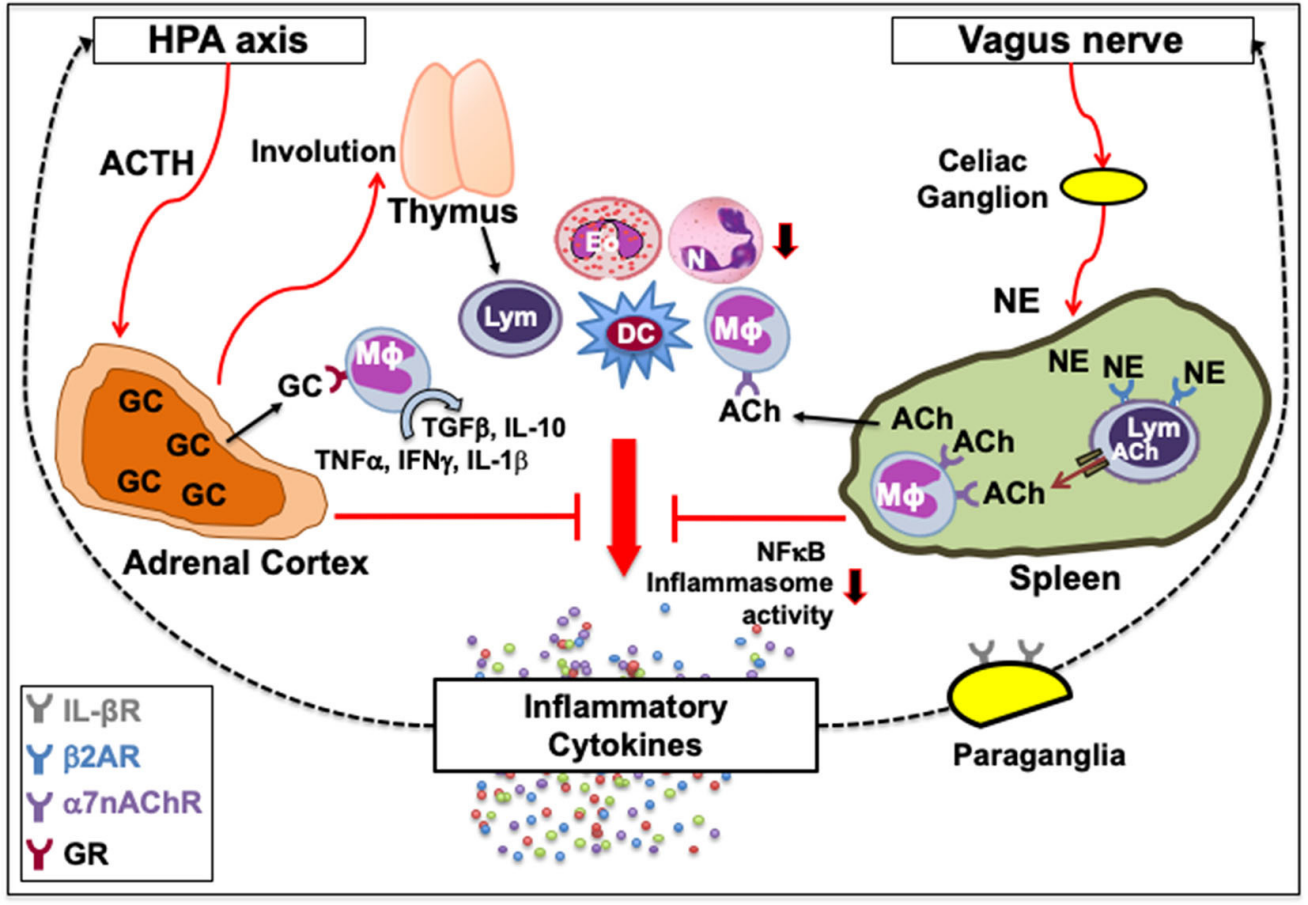
Classical neuroimmune network. [Left] A feedback circuit exists between inflammatory cytokines and the hypothalamic-pituitary-adrenal (HPA) axis. Upon activation of the HPA axis and the release of adrenocorticotropic hormone (ACTH) following various physiologic or pathologic stress, adrenal cortex synthesizes glucocorticoids (GC) that can affect immune function by causing thymic involution, neutrophil (*N*) mobilization, eosinophil (Eo) accumulation, and myeloid and granulocyte differentiation, or polarize immune cells from inflammatory to tissue-protective non-inflamed phenotypes. GC upon binding with their receptors (GR) on macrophages (Mϕ) can switch their inflammatory cytokine secretions, such as TNFα, IFNγ and IL-1β, to tissue-protective types such as TGFβ and IL-10. [Right] Vagus nerve forms a synapse at the celiac ganglion with the adrenergic splenic nerve, which comes in contact with lymphocytes (Lym) expressing β2 adrenergic receptor (β2AR). Norepinephrine (NE) released from the adrenergic splenic nerve terminals activates β2AR that facilitates the synthesis of acetylcholine (ACh) from lymphocytes (33, 41). The ACh thus released activates the cognate α7 nicotinic ACh receptors (α7nAChR) on intrasplenic or extrasplenic myeloid cells. Signal transduction following ACh-induced activation of α7nAChR decreases nuclear translocation of the nuclear factor κ-light-chain-enhancer of activated B cells (NFκB), and inhibits inflammasome activity to reduce the production of inflammatory cytokines. This neuronal reflex circuit through the afferent vagus nerve, called cholinergic anti-inflammatory pathway, can attenuate the exacerbated “non-resolving inflammation” by suppressing accumulation of neutrophils and acting on Mϕ, dendritic cells (DC) and lymphocytes. It is also suggested that IL-1β receptors are present on vagal paraganglial glomus cells that can release neurotransmitters in response to hypoxemia, hypercapnia or acidosis. Downward arrows indicate a decrease in activity or number. Dotted lines indicate a feedback loop.

The CNS is hardwired to monitor the presence of cytokines and molecular products of inflammation. IL-1β is a key pro-inflammatory cytokine in the inflammasome pathway that is secreted in response to any stress, including oxidative stress, and stimulates the HPA axis (44–47). In turn, adrenal cortex synthesizes glucocorticoids (GC) that can cause thymic involution, neutrophil mobilization, myeloid, and granulocyte differentiation and eosinophilia (48–52) or polarize immune cells from inflammatory to tissue-protective non-inflamed phenotypes (44, 45). GC upon binding with GC receptors (GR) on macrophages can switch their inflammatory cytokine secretions, such as TNFα, IFNγ, and IL-1β, to tissue-protective types such as TGFβ and IL-10. This promotes tissue repair by enhancing phagocytosis of cells undergoing apoptosis while preventing their necrosis (53, 54) (Figure 1, left). In addition, a cytokine storm of IL-1β causes hyperalgesia, a common characteristic of any sickness (55).

Another prominent link is between the vagus nerve and the immune system. The vagus nerve originates from the nucleus ambiguous and dorsal motor nucleus of the brainstem. In humans, it comprises 80,000–100,000 fibers as a major conduit for transmitting sensory information from most visceral organs to the brain (56). Vagus nerve forms a synapse at the celiac ganglion with the adrenergic splenic nerve, which comes in contact with lymphocytes expressing β2AR (57, 58). Norepinephrine (NE) released from the adrenergic splenic nerve terminals activates β2AR that facilitates the synthesis of acetylcholine (ACh) from lymphocytes (44, 59). The ACh thus released activates the cognate α7 nicotinic ACh receptors (α7nAChR) on neighboring monocytes and macrophages (44, 60, 61). Signal transduction following ACh-induced activation of α7nAChR increases intracellular Ca^2+^, decreases nuclear translocation of the nuclear factor κ-light-chain-enhancer of activated B cells (NFκB), stabilizes mitochondrial membranes, and inhibits inflammasome activity to reduce the production of inflammatory cytokines, such as TNFα, IL-1, IL-6, and IL-18 (62–65). Thus, activation of the vagus nerve attenuates the systemic inflammatory response by macrophages through the release of ACh (Figure 1, right). The cholinergic signaling is also known to suppress accumulation of neutrophils (66). This neuronal reflex circuit is called cholinergic anti-inflammatory pathway (58, 67). Neural signals through the afferent vagus nerve can attenuate the exacerbated “non-resolving inflammation” by acting on dendritic cells (DC) and lymphocytes (54, 60). It was recently shown that nerve impulses recorded from the vagus nerve of mice exposed to IL-1β and TNF exhibited distinct shape, amplitude, and firing rates, suggesting that the information present in afferent sensory signals within the vagus nerve can discriminate between specific cytokines (68). It is suggested that IL-1β receptors are present on vagal paraganglial glomus cells that act as secretory sensory neurons and release neurotransmitters in response to hypoxemia (low pO_2_), hypercapnia (high pCO_2_), or acidosis (low pH) (69, 70). Thus, the CNS and the immune system exchange information to act collectively in health and disease.

An extensive crosstalk between innate immune pathways and neuronal circuits has a bearing on human diseases (71). Likewise, adaptive T cells and cytokines associated with adaptive responses, such as IL-4, IFN-γ, and IL-17, have been implicated in neuroimmune crosstalk. IL-4 derived from CD4^+^ T cells present in the meninges has been shown to prevent the pro-inflammatory differentiation of resident meningeal myeloid cells in mice and induce the expression of brain-derived neurotrophic factor in astrocytes (72). IFNγ from meningeal T cells supports neuronal circuits that are associated with social behavior (73), whereas IFNγ from choroid plexus T cells has been associated with memory function and hippocampal neurogenesis (74–76). In addition, brain development during embryogenesis is influenced by cytokines derived from maternal adaptive immune cells. Maternal T_H17_ cells have been shown to induce structural alterations in the cortex and autism-like behavioral abnormalities (77).

## Immunomodulatory Neurotransmitters

Neuroimmune interaction depends on cell-to-cell contacts and on soluble communication molecules that include cytokines, chemokines, neuropeptides, neurotrophins, and neurotransmitters. Various leukocytes at different activation states synthesize neurotransmitters and express their receptors to participate in neuroimmunomodulatory circuitry (78–81). Similarly, neurons express pattern-recognition receptors (PRR), including TLRs and cytokine receptors, to influence immune pathways (82). This provides a common molecular platform for neuroimmune network for handling responses against obnoxious threats, including those triggered by PAMP (83–86). Macrophages, DC, T cells, and other immune cells express ionotropic, metabotropic, and G-protein-coupled receptors for various neurotransmitters (87–89). Besides ACh and NE, a variety of neurotransmitters, such as dopamine, serotonin, substance P, γ-aminobutyric acid (GABA), and glutamate modulate immune responses. While glutamate is the main excitatory neurotransmitter, GABA is the chief inhibitory neurotransmitter in the mature mammalian CNS. Outside the brain, GABA is produced by pancreatic β-cells as well as T cells and macrophages that express all components of GABAergic system, including its receptors, transporters, and metabolic enzymes. GABA acts as a negative regulator of macrophage and microglial production of inflammatory cytokines and T cell activation by blocking calcium signaling and NFκB activity (90). It is implicated in several autoimmune diseases, such as experimental autoimmune encephalomyelitis (EAE), multiple sclerosis (MS), inflammatory bowel disease (IBD), type-1 diabetes, arthritis, and dermatitis.

A review of the entire range of neurotransmitters involved in neuroimmune interaction is beyond the scope of this article. Prior reviews have covered the cholinergic (acetylcholine), catecholaminergic (norepinephrine and epinephrine), and vagal nerve regulation of immunity (65, 91). Detailed reviews are also available on the role of enteric nervous system in the regulation of immune responses in the gastrointestinal (GI) tract (92, 93), as well as on the HPA axis, a stress-responsive combinatorial axis of CNS and endocrine systems, with major immunoregulatory mechanism for fine-tuning the innate and adaptive immunity (94–97). GABAergic neuroimmune interactions have also been reviewed extensively (90, 98–100).

In the present article, we have reviewed the available knowledge on four critical neurotransmitters, namely, dopamine, serotonin, substance P, and glutamate that are known to be broadly present outside the nervous system. We discuss their effects on immune responses and potential clinical implications of their modulation. Activation of the neurotransmitter receptors on immune cells can result in the stimulation or suppression of myeloid and lymphoid cells through a variety of effects. In our discussion, any activation of neurotransmitter receptors leading to an enhanced immune response is categorized as an immunostimulatory effect irrespective of the disease. This includes the situation where the decreased suppressive activity of an immune cell, such as T_reg_, results in an overall augmented immune effector function. By contrast, an activation of these receptors leading to a reduced immune cell development, differentiation, numbers, function, increased immune cell death, dysfunction, or tolerance is categorized as an immunosuppressive effect.

### Dopamine

Dopamine or 3,4-dihydroxyphenethylamine (DA) belongs to the catecholamine and phenethylamine families. It is found in plants with the highest concentrations observed in bananas (101), and in most multicellular animals, including cnidarians (102). This dates the emergence of DA as a neurotransmitter to the earliest appearance of the nervous system, over 500 million years ago in the Cambrian Period (103). It is synthesized from L-tyrosine by tyrosine hydroxylase in the ventral tegmental area and the substantial nigra pars compacta neurons of the CNS that innervates primary and secondary lymphoid organs (104–106). In the brain, DA functions as a major player in the motivational component of reward behavior and regulates arousal and mood besides controlling motor movement. Across a wide range of vertebrates, DA spike triggers behavior-switching and response selection that confers motivational salience for an organism's growth and survival (102, 107).

**Figure 2:**
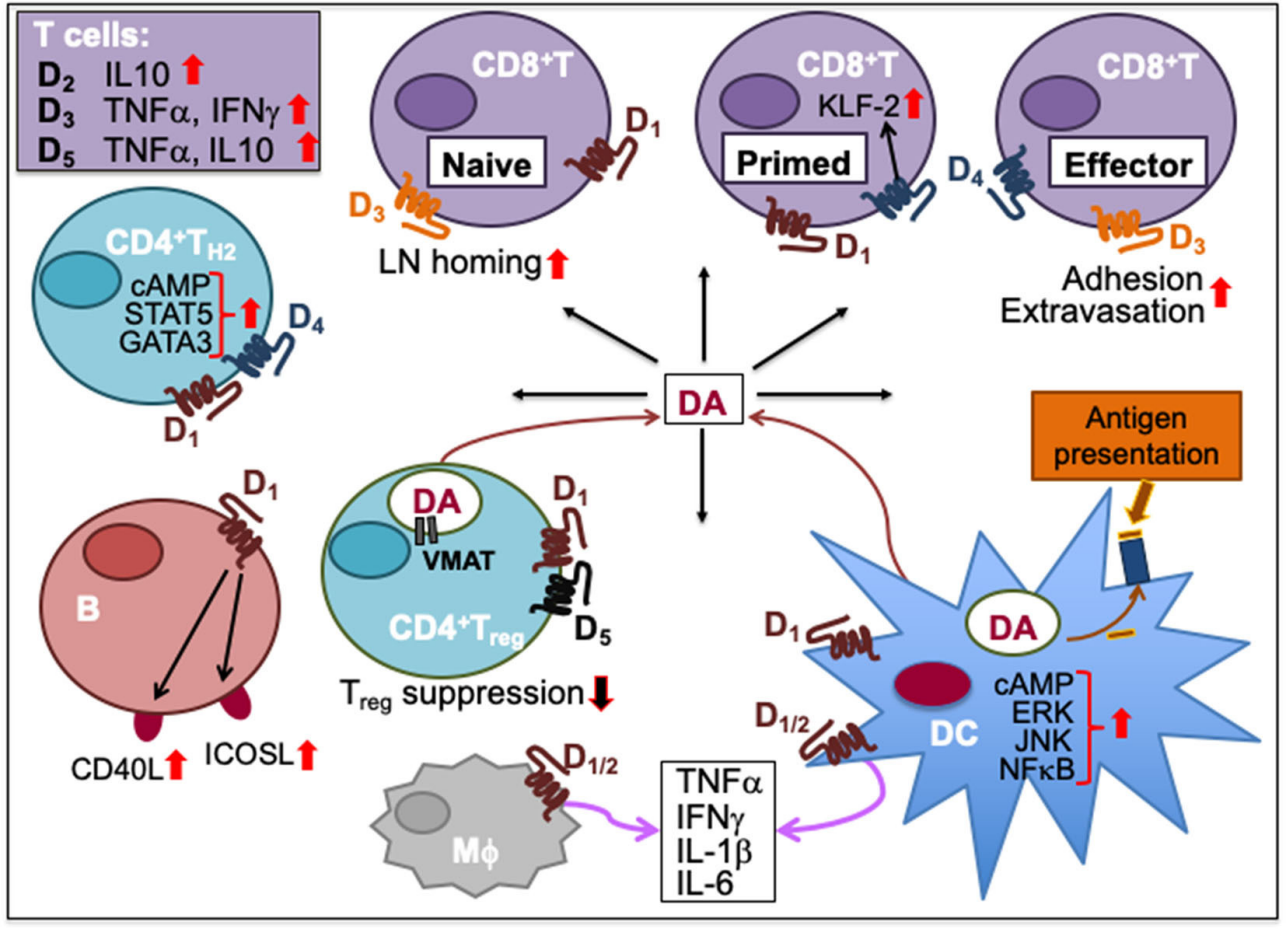
Dopamine-mediated cell-to-cell communication among immune cells. Dopamine (DA) can induce cytokine secretion and influence immune responses through activation of dopamine receptors, D_1-5_. D_2_ activation increases IL-10, D_3_ activation triggers T cell secretion of TNFα and IFNγ, and D_5_ activation stimulates TNFα and IL-10 secretion in T cells. D_1_ and D_4_ activation promotes T_H2_ differentiation by increasing cAMP and transcription factor STAT5 and GATA3 activity. D_1_ activation on B cells increases their expression of inducible T cell costimulatory ligand (ICOSL) and CD40L. Activation of D_1_ and D_5_ on regulatory T (T_reg_) cells reduces their suppressive activity. Helper T (T_H_) cells including T_reg_ cells can synthesize DA, store it in their vesicles expressing vesicular monoamine transporter (VMAT) and release it upon activation. Activation of D_3_ on naïve CD8^+^T cells triggers their chemotactic migration. D_3_ activation also facilitates effector CD8^+^T cell extravasation and adhesion to fibronectin. D_4_ activation on primed CD8^+^T cells increases Krüpple-like factor-2 (KLF-2) activity to cause CD8^+^T cell quiescence. Activation of D_1_ or D_2_ receptors expressed on both macrophages (Mϕ) and dendritic cells (DC) promotes the secretion of cytokines such as IFNγ, TNFα, IL-1β, and IL-6. D_1_ activation on DC promotes ERK, JNK and NFκB signaling, which induces cytokine production. D_1_ activation also induces DC synthesis of DA during antigen presentation to T cells, consequently activating D_1_ on CD8^+^T cells. Upward solid red arrows depict upregulation of indicated molecules or effects whereas the downward solid arrows indicate the reverse. Thin red line arrows indicate cells that produce DA.

Outside the CNS, DA functions as an exocrine or paracrine messenger (108). In the immune system, it affects leukocytes in the LN, spleen, bone marrow, and circulation (109). Immune cells such as activated T_H_, T_reg_ and mature DC express tyrosine hydroxylase that synthesize DA, and store it in vesicles expressing vesicular monoamine transporter subtype 1/2 (VMAT1/2) (110–112). Moreover, all five subtypes of DA receptors (DR), namely, D_1_-D_5_, are expressed in immune cells. DRs are metabotropic G-protein-coupled receptors (GPCR), meaning that they exert effects via a second messenger system. DRs form a heteromeric network with a number of other GPCRs such as adenosine A_2A_ (113, 114). D_1_ and D_5_ (D_1_-like family) activate Gα that increases intracellular cyclic adenosine monophosphate (cAMP) while D_2_, D_3_, and D_4_ (D_2_-like family) inhibit adenylyl cyclase and reduce cAMP levels via Gαi signaling (115). D_5_ was the first DR shown to be expressed in human peripheral blood lymphocytes (116). Human B and T cells exhibit minimal to moderate levels of D_2−5_ expression (117). T_reg_ and T_H17_ cells express D_1_ (110, 118). Natural killer (NK) cells express all DRs except D_1_ while DCs express all subtypes (117, 118). DA effect on a target cell depends on the types of DRs and the intracellular response to the second messengers. In neurons, the ultimate effect of D_1_ family receptor (D_1_ and D_5_) activation can be excitatory (via opening of Na^+^ channels) or inhibitory (via opening of K^+^ channels); the ultimate effect of D_2_ family (D_2_, D_3_, and D_4_) activation is usually inhibitory. Various effects of dopamine on myeloid and lymphoid immune cells are summarized in Table 1. Based on the available information, the immune network of dopamine-mediated cell-to-cell communication is depicted in Figure 2.

**Table 1:** Summary of the effects of dopamine on myeloid and lymphoid immune cell responses.

**Neurotransmitter**	**Receptors**	**Target immune cells**	**Implications in disease**
**Dopamine** 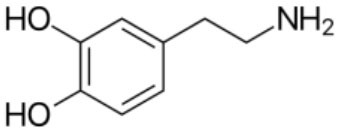	D_1_ D_2_ D_3_ D_4_ D_5_	CD4^+^T CD8^+^T T_reg_ T_FH_ NK DC	Colitis IBD Multiple sclerosis Schizophrenia Alzheimer's disease Parkinson's disease
**Cellular adhesion and homing**			
D_3_ activation promotes homing of naïve T cells to lymph nodes (105) D_3_ activation induces T cell adhesion to fibronectin and ICAM (103, 119, 120) D_1_ activation downregulates Treg adhesion and migration (121)			
**Lymphocyte function**			
T_reg_, T_FH_, and activated T_H_ cells express tyrosine hydroxylase and synthesize DA (110–112, 122) D_1/5_ activation downregulates T_reg_ suppressive, adhesive, and migratory abilities (121, 123) D_1/4_ activation promotes T_H2_ and T_H17_ differentiation (112, 124) D_1_ activation increases B cell expression of ICOSL and CD40L (122) D_2_ activation increases IL-10, D3 activation triggers TNFα and IFNγ and D5 facilitates TNFα and IL-10 secretions in T cells (125) D_4_ activation increases KLF-2 expression, inhibits ERK1/2 and Lck and Fyn in CD8^+^T cells and facilitates their quiescence (126–129) D_5_ activation suppresses NK cell proliferation and IFNγ production (130) DA increases ROS production in peripheral blood lymphocytes in an autocrine manner (126, 131)			
**Dendritic cell maturation, antigen presentation, and myeloid cell function**			
D_1_ activation increases DC ERK, JNK, and NFκB activity (132) D_1_-like activation triggers DC tyrosine hydroxylase activity and DA production during antigen presentation (112) D_1/2_ activation increases macrophage and DC secretion of INFγ, TNFα, IL-6, and IL-1β (133) D_2_ activation inhibits DC adenylyl cyclase activity (132)			

*T_reg_, Regulatory T cells; T_FH_, Follicular helper T cells; NK, Natural killer cells; DC, Dendritic cells; ICAM, Intercellular adhesion molecule; DA, Dopamine; ICOSL, Inducible T cell costimulatory ligand; CD40L, CD40 ligand; KLF-2, Krüpple-like factor-2; ERK, Extracellular signal-regulated kinases; Lck, Lymphocyte-specific protein tyrosine kinase; Fyn, Proto-oncogene tyrosine-protein kinase; JNK, c-Jun N-terminal kinase; NFκB, Nuclear factor κ-light-chain-enhancer of activated B cells; IBD, Inflammatory bowel disease; ROS, Reactive oxygen species*.

#### Immunostimulatory Effects

In immune cells, DA signaling through specific DRs can enhance activation and stimulate cytokine secretion. D_2_ activation triggers human T cell secretion of IL-10, D_3_ activation facilitates TNFα and IFNγ, and D_5_ facilitates TNFα and IL-10 secretions (125). Time kinetics differs for the D_5_ stimulation of TNFα, an inflammatory cytokine characteristic of T_H1_ response, and IL-10, an anti-inflammatory cytokine characteristic of T_H2_. DA-induced TNFα secretion occurred at 24 h while IL-10 secretion occurred at 72 h following activation in human T cells (104, 125). Hence, immune effects mediated by DA may be first inflammatory T_H1_-type, then switching to anti-inflammatory T_H2_-type following activation.

Recently, it was shown that sympathetic nerves undergo a dopaminergic-to-adrenergic transition during post-natal development of the lung in mice and humans. DA signaling through D_4_ promotes T_H2_ differentiation. The DA:D_4_ signaling acts synergistically with IL-4 by upregulating IL-2Rα-STAT5 pathway and reducing inhibitory histone trimethylation at T_H2_ gene loci of GATA-3 (124). This mechanism became marginal after sympathetic nerves turned adrenergic in the adult lung. Thus, the communication between dopaminergic nerves and T_H2_ cells underlies the susceptibility of post-natal lung to hypersensitivity reactions by coordinating release of IL-4 and histamine (which sensitize nociceptors), production of mucus and IgE antibodies (airway irritancy) and cough behavior.

D_1_ activation on DCs increased extracellular signal-regulated kinases (ERK), c-Jun N-terminal kinase (JNK) and NFκB activity that facilitated cytokine production and promoted T_H2_ and T_H17_ differentiation in humans and mouse models (112, 118, 132). D_1_ activation also stimulated DC tyrosine hydroxylase activity for the synthesis of DA during antigen presentation to T cells, consequently activating D_1_ on primed T_H_ cells. The increased DA binding with D_1_ on primed T_H_ cells increased their intracellular levels of cAMP, inducing T_H2_ polarization (112). Conversely, D_1/2_ antagonist berberine reduces the secretion of LPS-induced cytokines such as INF-γ, TNF-α, IL-6, and IL-1β from murine macrophages and DCs (133).

On the other hand, D_1_ activation on T_reg_ cells downregulate their migratory, adhesive, and suppressive abilities, which in turn result in an augmented effector function (121). In addition, human follicular T_H_ (T_FH_) cells can synthesize and release DA that upon binding with D_1_ on B cells increases their expression of inducible T cell costimulatory ligand (ICOSL) and CD40L and improves their survival (122). This DA-mediated mechanism may offer an important advantage for the control of infections by facilitating T_FH_-B cell synapses (122).

D_3_ activation triggers chemotactic migration in human and murine naïve CD8^+^T cells (105). It facilitates CD8^+^T cell β1 integrin binding to fibronectin and intercellular adhesion molecule (ICAM) of the endothelium allowing extravasation of T cells to their target sites (105, 119, 120). Indeed, a D_3_ antagonist U-99194-A inhibited the homing of naïve CD8^+^T cells to the inguinal LN in mice (105). Thus, a collaborative DA-mediated network of DC, T_H_, and B cells along with decreased T_reg_ suppressive activity could be helpful in priming and sustaining CD8^+^T cell cytolytic responses against parasitic infections and in immunosuppressive conditions.

#### Immunosuppressive Effects

DA signaling through D_4_ is known to suppress lymphocyte function by attenuating secretion of IL-2 and IFNγ in human T cells through upregulation of master transcription factor Krüpple-like factor (KLF-2) and inhibition of ERK1/2 kinases (126–128). DA inhibits the expression of lymphocyte-specific protein tyrosine kinases, Lck, and Fyn, which are critical for TCR signal transduction (127). Under *in vitro* conditions of elevated physiological concentrations of DA, CD8^+^ T cells were found to be more susceptible to D_1_ family receptor-mediated inhibition of proliferation and downregulation of cytolytic activity relative to CD4^+^ T cells (129). Activation of D_4_ facilitated quiescence of activated T cells through the expression of KLF-2 (128). Moreover, DA released from T cells can result in enhanced production of intracellular reactive oxygen species leading to oxidative stress and apoptosis in human peripheral blood lymphocytes in an autocrine mechanism to downregulate their own proliferation and differentiation (126, 131). D_5_ activation by DA on NK cells suppressed their proliferation and IFNγ production without affecting apoptosis or cell death (130). In a mouse model of septic polymicrobial peritonitis, the stimulation of sciatic nerve inhibited the production of TNF, monocyte chemotactic protein-1, IL-6, and INFγ induced by LPS via vagal stimulation and release of DA activating D_1_ receptors on myeloid cells (134). In DCs, D_2_ activation inhibited adenylyl cyclase activity and reduced intracellular levels of cAMP, which in turn impaired T cell proliferation and cytokine production (132). Overall, it appears that by selectively triggering specific DR on immune cells DA may provide for an immunological behavior-switching and context-dependent immunostimulatory or immunoinhibitory response selection for an organism's survival.

#### Clinical Implications

Several autoimmune diseases have been linked to DA modulation. In an experimental model of colitis, administration of berberine diminished inflammation in the colon by reducing the levels of IFN-γ and IL-17 secretions from colonic macrophages (135). In IBD, dopaminergic system is responsible for altered T cell signaling leading to augmented effector function and decreased suppressive role of T_reg_ via D_5_ (123). Patients with MS downregulate D_5_ on T cells with inability to reduce proliferation or IFN-γ secretion when DA is administered, suggesting a dysregulated DA function in MS pathology (136). In relapsing MS patients, suppressive function of T_reg_ was restored by diminishing the effects of DA on T cells after IFN-β treatment (123). Also, administration of a D_1_ antagonist SCH-23390 downregulated IL-6–dependent T_H17_ response (137).

The impaired immune responses by DA is also linked to a number of brain disorders, such as schizophrenia (138), Alzheimer's disease (AD) (139, 140) and Parkinson's disease (PD) (141). Besides microglia, the brain-specific macrophages, CNS-infiltrating lymphocytes play a significant role in these brain disorders. Schizophrenia patients express more D_3_ (142) whereas patients with AD express lower D_2_ on T cells compared to healthy controls (143). The progression of PD is dependent on CD4^+^T, not CD8^+^T, cell infiltration into the substantia nigra as well as loss of dopaminergic neurons of the nigrostriatal pathway (123, 144, 145). One study demonstrated that L-DOPA or a D_2_ agonist bromocriptine administration to PD patients attenuated the increased expression of D_1_ and D_2_ on peripheral blood lymphocytes (146). It is also postulated that PD may originate from an autoimmune mechanism involving IgG antibodies that bind to DA in neurons inducing their death (147, 148). In a PD-inducing neurotoxin 1-methyl-4-phenyl-1,2,3,6-tetrahydropyridine mouse model, dopaminergic cell death was almost exclusively caused by the substantia nigra-infiltrating CD4^+^T cells by a FasL-mediated cytolytic pathway, but not by IFNγ-dependent mechanisms, in an environment replete with activated microglia and pro-inflammatory cytokines (144, 149, 150).

Thus, DA modulation may offer treatment alternatives to mitigate neuropathologies and autoimmune diseases. Based on few studies discussed above, DA agonists may help in brain disorders such as PD whereas specific DR antagonists could help alleviate MS, schizophrenia, AD, colitis, and IBD.

### Serotonin

Serotonin or 5-hydroxytryptamine (5-HT) is found in all bilateral animals including worms and insects (151), as well as in fungi and plants with the highest values present in nuts of walnut and hickory (152). In the brain, it is synthesized in serotonergic neurons in the Raphe nuclei located in the brainstem, and mediates various behavioral processes (153–155). However, neuronal 5-HT consists merely 10% of the 5-HT produced in the body. Majority of 5-HT is synthesized by enterochromaffin cells of the GI tract (156, 157), where it mainly regulates intestinal movements besides controlling bone mass and endothelial nitric oxide synthase activity (158). A GI parasite *Entamoeba histolytica* becomes more virulent in the presence of high levels of 5-HT in the gut, and secretes additional 5-HT to thrive by a phenomenon known as quorum sensing (159) (similar to quorum sensing in the regeneration of distressed hair follicles on skin in the presence of CCL2 and TNFα. The concentration of 5-HT and the strength of its signaling mediate brain's perception of resource availability. In less complex animals, such as invertebrates, resources simply mean food availability (160, 161). In more complex arthropods and vertebrates, resources also can mean social dominance (162, 163). 5-HT spikes may elevate or lower an organism's mood and behavior in response to the perceived abundance or scarcity of resources for its social growth and survival.

5-HT synthesis from enterochromaffin cells receiving short-chain fatty acids from the gut microbiota is transported in the bloodstream for uptake by innate and adaptive immune cells via membrane-expressing serotonin transporters (SERT) including platelets, which store it in granules (164, 165). Whenever platelets bind to the endothelial lesion in damaged tissue during blood clotting, 5-HT acts as a vasoconstrictor to stop bleeding, and as a fibrocyte mitotic factor to aid tissue-healing. In response to inflammation, mast cells, macrophages, and T cells can produce 5-HT on their own by upregulating the expression of tryptophan hydroxylase 1 and 2 enzymes required to synthesize 5-HT from 5-hydroxytryptophan (87, 166, 167).

**Figure 3:**
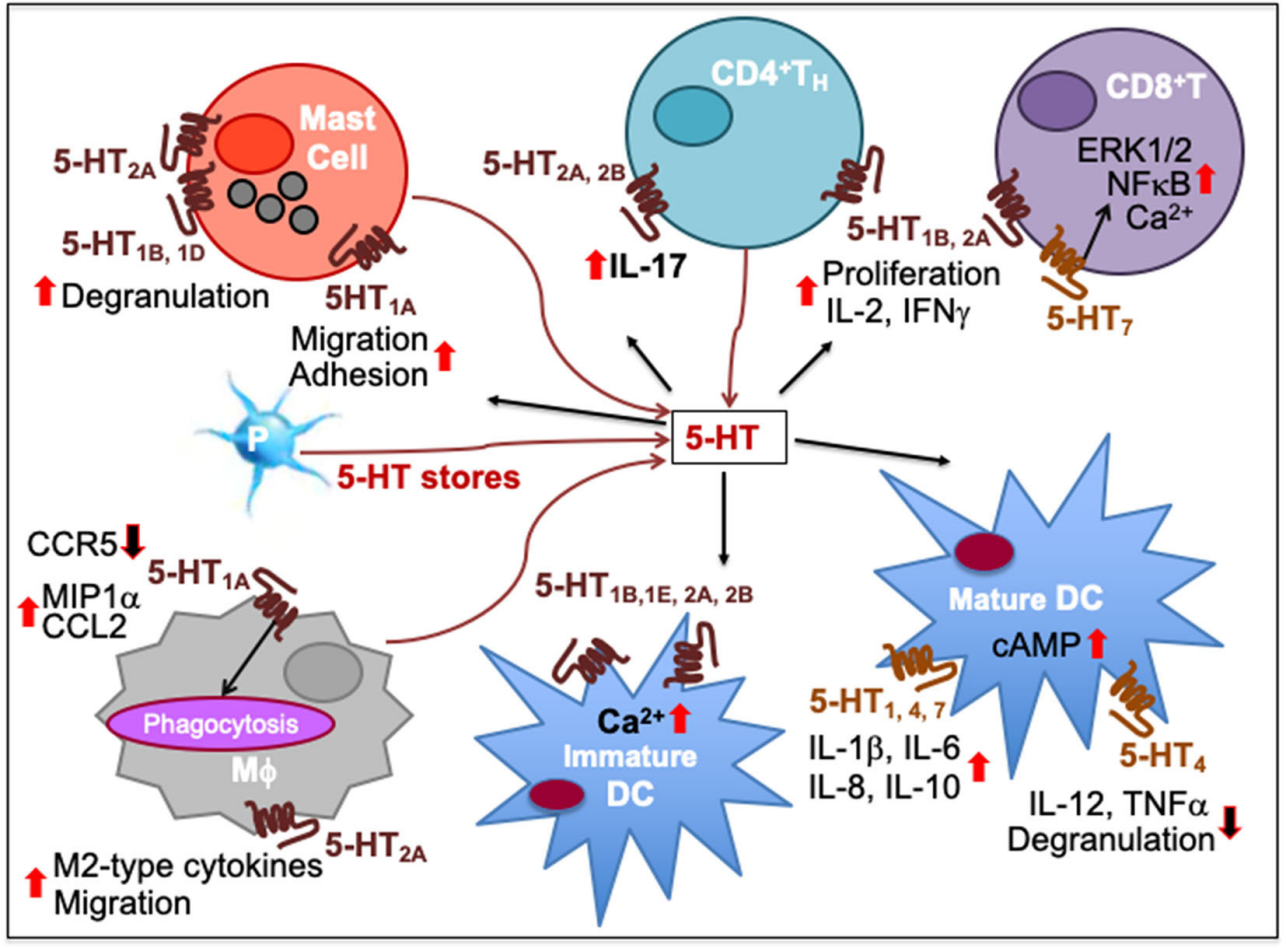
Immune network of serotonin-mediated cell-to-cell communication. In mast cells, 5-HT_1A_ activation increases their migration and adhesion while 5-HT_1B_, 5-HT_1D_, and 5-HT_2A_ induces their degranulation and activity. 5-HT released from platelet (*P*) stores act upon myeloid and lymphoid cell subsets during inflammatory processes. As T cells differentiate, more 5-HT_7_ are expressed together with 5-HT_1B_ and 5-HT_2A_, increasing intracellular Ca^2+^. 5-HT_7_ activation facilitates T cell proliferation through ERK1/2 phosphorylation and NFκB activation. 5-HT_1B_ and 5-HT_2A_ activation promotes T cell production of IL-2 and IFNγ and antigen-specific proliferation in T_H1_ and CD8^+^ cytotoxic T lymphocytes (CTL). 5-HT_2A_ and 5-HT_2B_ activation promotes IL-17 production from CD4^+^T cells. 5-HT_1A_ activation has been implicated in decrease of CCR5, increase of CCL2 and MIP1α as well as phagocytosis by macrophages (Mϕ) while 5-HT_2A_ activation increases production of M2-type cytokines and migration. 5-HT signaling through 5-HT_1B_, 5-HT_1E_, 5-HT_2A_, and 5-HT_2B_ increases intracellular Ca^2+^ in immature DCs. It also enhances DC maturation by upregulating cAMP levels through 5-HT_4_ and 5-HT_7_. Moreover, 5-HT_1_, 5-HT_4_, and 5-HT_7_ activation on mature DCs promotes secretion of cytokines, such as IL-1β, IL-6, IL-8, and IL-10 whereas 5-HT_4_ inhibits DC degranulation and production of IL-12 and TNFα. Upward solid red arrows depict upregulation of indicated molecules or effects whereas the downward solid arrows indicate the reverse. Thin red line arrows indicate cells that produce 5-HT.

Most immune cells express 5-HT receptors (5-HTR) on their membranes (168). There are 14 subtypes of 5-HTRs in vertebrate animals and all but 5-HT_3_ are GPCR. While 5-HT_1_ and 5-HT_5_ are coupled to G_α*i*/*o*_ proteins that inhibit adenylyl cyclase, 5-HT_4_, 5-HT_6_, and 5-HT_7_ are coupled to G_α*s*_ proteins that activate adenylyl cyclase for cAMP production. 5-HT_2_ is coupled to G_q/11_ that activates phospholipase C-β, which facilitates the release of diacyl glycerol and formation of inositol 1,4,5-trisphosphate (IP3) (169, 170). Unlike all of these, 5-HT_3_ is a ligand-gated ion channel capable of transporting Na^+^ and K^+^ cations (168). Immune cells exhibit varied expression of these receptors. Microglia, the brain-resident macrophages, express 5-HT_2B_, 5-HT_5A_, and 5-HT_7_ receptors (171). 5-HT_1A_ is expressed in mast cells (172) and macrophages (167); 5-HT_1B_ is expressed in DC (169, 173); 5-HT_1E_ in monocytes (174) and DC (169); 5-HT_2A_ in eosinophils (175) and macrophages (176); and 5-HT_4_ and 5-HT_7_ in DC (169, 173). Among adaptive immune cells 5-HT_1B_, 5-HT_2A_, and 5-HT_7_ are expressed in T cells (177, 178), and 5-HT_3_ in both pre-B, mature and neoplastic B cells (179–181). Most of these 5-HT receptors impact activation or inhibition of immune responses (164). 5-HT can act both as a pro-inflammatory or anti-inflammatory tissue protective signaling molecule as observed in the intestinal mucosa via activation of 5-HT_7_ or 5-HT_4_ receptors, respectively (157). Various effects of 5-HT-triggered signaling on lymphoid and non-lymphoid cells are summarized in Table 2, and the immune network of 5-HT-mediated cell-to-cell communication is depicted in Figure 3.

**Table 2:** Summary of the effects of serotonin on immune cell responses.

**Neurotransmitter**	**Receptors**	**Target immune cells**	**Implications in disease**
**Serotonin** 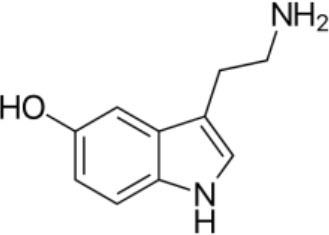	5-HT1A,B,D,E,F 5-HT2A,B,C 5-HT3 5-HT4 5-HT5A,B 5-HT6 5-HT7	Eosinophil Mast cell T cell DC	Allergic Asthma IBD Crohn's disease Ulcerative colitis Multiple sclerosis Cancer
**Cellular adhesion and homing**			
5-HT chemoattracts eosinophils and mast cells (164, 175) 5-HT induces neutrophil recruitment (169) 5-HT_1A_ signaling promotes bone marrow-derived mast cell migration and adhesion (172) 5-HT_2A_ activation promotes macrophage migration (182) 5-HT_2A_ agonist prevents eosinophil recruitment (183, 184)			
**Lymphocyte function**			
5-HT_7_ on naïve T cells facilitates their IL-2 secretion as well as activation and proliferation through increase in Ca^2+^ and ERK1/2 and IκBα phosphorylation (87, 164) 5-HT_1B_ and 5-HT_2A_ promote the production of IL-2 and IFNγ and the antigen-specific proliferation of T_H1_ and CD8^+^T cells (177, 178) 5-HT_2A_ and 5-HT_2B_ increases IL-17 production in CD4^+^T cells (185) Increases IL-10 production by T_H2_ cells (186) 5-HT favors expansion and function of FoxP3^+^CD39^+^ T_reg_ cells from MS patients (186) 5-HT_1_-like receptor activation increased K^+^ conductance in pre-B cells (181) 5-HT_3_ activation decreased K^+^ conductance and pre-B cell activation (181)			
**Dendritic cell maturation, antigen presentation, and non-lymphoid cell function**			
Platelets store and release 5-HT upon issue injury and inflammation (164) 5-HT_1B/1D_ and 5-HT_2A_ activation increases mast cell degranulation and activity (187, 188) DCs express SERT and shuttle serotonin to T cells during antigen presentation (164) 5-HT_1B_, 5-HT_1E_, and 5-HT_2A/2B_ stimulation induces intracellular Ca^2+^ mobilization via G_i/o_ proteins in immature DC (169, 173, 189) 5-HT_1_, 5-HT_4_, and 5-HT_7_ activation elevates cAMP in mature DC and microglial MC-3 cells, and enhances their secretion of IL-1β, IL-6, IL-8, and IL-10 (169, 173, 189) 5-HT_4_ activation reduces IL-12 and TNFα secretion and inhibits degranulation in mature DC (169) 5-HT_1A_ activation on macrophages stimulates secretion of chemokine CCL2 and phagocytosis (190) 5-HT_2A_ activation stimulates NFκB-mediated macrophage activity (182) 5-HT_1A_ agonist decreases the expression of CCR5 and increases the secretion of its ligand macrophage inflammatory protein-1α (191) 5-HT_2A_ agonist inhibits expression of TNFα-mediated migratory and inflammatory molecules (183, 184)			

*T_reg_, Regulatory T cells; DC, Dendritic cells; ERK, Extracellular signal-regulated kinases; cAMP, Cyclic adenosine monophosphate; SERT, serotonin transporter*.

#### Immunostimulatory Effects

Expression of 5-HT_7_ on naïve T cells is enhanced upon activation. In addition, activated T cells acquire the expression of 5-HT_1B_ and 5-HT_2A_, and synthesize 5-HT in an autocrine manner. Moreover, 5-HT_7_ activation induces IL-2 secretion and T cell proliferation through rapid phosphorylation of ERK1/2 and IκBα (87, 164). 5-HT_1B_ and 5-HT_2A_ further promote the antigen-specific proliferation of human and murine T_H1_ and cytotoxic CD8^+^T cells. Selective 5-HT_1B_ antagonist SB-216641 and 5-HT_2A_ inhibitor sarpogrelate hydrochloride inhibited T cell production of IL2 and IFNγ (177, 178). Also, exogenous 5-HT increased IL-10 production by T_H2_ cells *ex vivo* from MS patients (186). Thus, 5-HT may function as an intrinsic autocrine cofactor in T cell activation and function.

Tissue injury and inflammation stimulates the release of stored 5-HT from platelets and mast cells. During post-burn trauma, increased tissue 5-HT concentration supports wound healing as shown by an increased frequency of F4/80 macrophages in response to excisional punch biopsy on skin from mouse models (192). Moreover, under *in vitro* conditions murine peritoneal macrophages showed increased secretion of chemokine CCL2 and phagocytosis following 5-HT_1A_ activation by its agonist (7)-8-hydroxy-2-(di-n-propyl-amino)-tetralin (190). Also, in mouse models of experimental colitis 5-HT_2A_ blockade by ketanserin impaired macrophage migration and decreased the NFκB-mediated activity (182). 5-HT was found to stimulate different signaling pathways in human T cells depending on their stage of maturation. Using isotype-selective receptor agonists, it was revealed that 5-HT_1B_, 5-HT_1E_, and 5-HT_2_ stimulation induced intracellular Ca^2+^ mobilization via G_i/o_ proteins in immature, but not mature, DC. Furthermore, functional studies indicated that activation of 5-HT_1_, 5-HT_4_, and 5-HT_7_ elevated cAMP in mature human DC and microglial MC-3 cells, and enhanced their secretion of IL-1β, IL-6, IL-8, and IL-10 (169, 173, 189). 5-HT_1A_ signaling played a role in the adhesion of bone marrow-derived mast cells as ${5-\text{HT}}_{1\text{A}}^{-/-}$ mice showed deficiency in their migration and adhesion (172). Also, 5-HT_1B/1D_ receptor agonist sumatriptan increased the number of degranulated mast cells in the brain thalamus (187), and 5-HT_2A_ activation increased endotoxin-induced mast cell activity in rats (188).

#### Immunosuppressive Effects

5-HT signaling has inhibitory effects especially on the pro-inflammatory activation of immune cells. It is well-accepted that many SERT blockers including the serotonin selective reuptake inhibitors (SSRI), which increase extracellular 5-HT concentration, have immunoinhibitory effects, such as reduced lymphocyte proliferation and decreased cytokine production by myeloid cells in both animals and humans (193–196). In a meta-analysis study of 827 patients suffering with major depressive disorder (MDD), the pooled effect estimate using bias-corrected standardized mean difference indicates that SSRI treatment decreases serum levels of pro-inflammatory cytokines, IL-6, TNFα, and IL-1β, and anti-inflammatory cytokine IL-10 (194, 197–199). In a murine LPS-induced septic shock model, fluoxetine markedly decreased circulating levels of TNFα (200). In a model of ovalbumin-sensitized allergic asthma, fluoxetine decreased monocyte-production of TNFα concomitant with decreased lung inflammation by reducing the number of macrophages, lymphocytes, neutrophils, and eosinophils (200). In this allergic asthma model, inhalation of 5-HT_2A_ agonist (R)-2,5-dimethoxy-4-iodoamphetamine [(R)-DOI] prevents the development of mucus hyperproduction, airways inflammation, and pulmonary eosinophil recruitment. Further, agonistic activation of 5-HT_2A_ inhibits the expression of a variety of TNFα-mediated migratory and inflammatory molecules, such as ICAM-1, VCAM-1, IL6, and NO synthase and NFκB activity in murine peripheral tissues including primary aortic smooth muscle cells, vasculature, and gut (183, 184). Other SSRIs such as venlafaxine and moclobemide reduced the antigen-specific humoral and delayed hypersensitivity T_H1_ cell-mediated immunity in mice with a reduction of the release of macrophage pro-inflammatory mediators and the expression of antigen-presentation markers (201). This suggests that SSRIs may owe some of their therapeutic effects to alleviation of the chronic inflammation. Also, exogenous 5-HT reduced *ex vivo* production of T_H1_ and T_H17_ cytokines from patients with MS, a demyelinating autoimmune disease mediated by T_H1_ and T_H17_ cells. Additionally, 5-HT reduced IFNγ and IL-17 secretion by CD8^+^T cells, enhanced T_reg_ function, and favored the expansion of FoxP3^+^CD39^+^ T_reg_ and T_reg17_ cells, a novel T_reg_ subset from MS patients (186).

Ionic permeability on voltage-gated K^+^ channel is also regulated by 5-HT signaling as demonstrated in murine pre-B cells. Using specific receptor antagonists, it was found that K^+^ conductance increased via the activation of 5-HT_1_-like receptors, whereas it decreased via 5-HT_3_ activation in pre-B cells. Moreover, 5-HT_3_ agonist, 2-methyl-5-HT accelerated the inactivation of pre-B cells (181), suggesting that B cell activation may be deregulated by 5-HT_3_, a ligand-gated cation channel (202). In mature DC, the activation of 5-HT_4_ reduces the secretion of IL-12 and TNF-α, and 5-HT_4_ agonist tandospirone citrate inhibited their degranulation (169). One study also demonstrated that 5-HT or 5-HT_1A_ agonist reduced HIV-1 infection in primary culture of human macrophages by decreasing the expression of CCR5 and increasing the secretion of its ligand macrophage inflammatory protein-1α on macrophages (191).

#### Clinical Implications

TNFα-mediated inflammatory pathways have been strongly implicated in several diseases, including atherosclerosis, rheumatoid arthritis, psoriasis, type II diabetes, depression, schizophrenia, and AD. Activation of 5-HT_2A_ represents a potential therapeutic avenue for the treatment of such disorders. Moreover, the inhibitory effects of 5-HT_2A_ agonist (R)-DOI on TNFα-mediated inflammation last many hours after the administration of TNFα (184). Thus, 5-HT-agonist-based therapies can prevent inflammation and may also treat inflammation-associated tissue stress or injury that has already occurred or is ongoing.

MDD is also heavily linked to systemic inflammatory responses through 5-HT (194, 195, 203–205). SSRIs alleviate depressive symptoms by decreasing the myeloid cell production of pro-inflammatory cytokines, resisting mast cell activation, and reducing chronic inflammation (193–196). MS patients with depression characterized with a high concentration of pro-inflammatory T_H1_ and T_H17_ cytokines are related to reduced synthesis and availability of 5-HT in the CNS (206). The *in vitro* treatment of PBMCs from MS patients with exogenous 5-HT reduced the production of T_H1_ and T_H17_ cytokines as well as increased the frequency of T_reg_, suggesting a critical role of 5-HT in downregulating an exacerbated inflammatory response in MS (186).

Dysregulation in 5-HT signaling affects multiple peripheral organs, such as the lung, heart, GI tract, genitourinary tract, and liver (158). The GI enterochromaffin cells synthesize 5-HT for release into the bloodstream linking it to several GI disorders, including irritable bowel syndrome (IBS) and constipation (157, 207, 208). SERT-expressing platelets containing 5-HT stores also affect gut inflammation that underlies GI disorders (209–211). A mouse model of colitis showed increased levels of 5-HT and IL-13 in the gut (212). Patients diagnosed with IBS or other bowel diseases show high levels of 5-HT and an increased frequency of T_H17_ cells. In fact, both 5-HTR agonists and antagonists have been used to treat IBS that also helped with other related symptoms (213). 5-HT_3_, 5-HT_4_, and 5HT_1B_ are the most important receptors for GI function. 5-HT_4_ agonists (e.g., tegaserod) potentiate peristalsis initiated by 5-HT_1_ stimulation and are thus helpful in chronic constipation and constipation-predominant form of IBS. 5-HT_3_ antagonists (alosetron and cilansetron) prevent the activation of 5-HT_3_ on extrinsic afferent neurons and can decrease the visceral pain associated with IBS.

In addition, studies have shown that 5-HT may alleviate symptoms associated with asthma, such as severe bronchoconstriction, mucus buildup, and eosinophil-induced inflammation in response to allergens (169, 175). In rheumatoid arthritis (RA) analysis of patients and murine models show that 5-HT is responsible for balancing T_H17_/T_reg_ populations (214). In a RA mouse model, the absence of 5-HT led to conversion of T_reg_ into T_H17_ cells, skewing the population to more pro-inflammatory type and worsening of tissue damage (185). In collagen-induced arthritis in Tph1^−/−^ mice, which have markedly reduced peripheral serotonin levels, *ex vivo* serotonin and 5-HT_2A_ and 5-HT_2B_ agonists restored IL-17 secretion from splenocytes and T_H17_ cell differentiation (185). Also, mast cell production of 5-HT worsened thrombocytopenia in dengue virus infections through platelet activation via 5-HT_1_ (215).

Thus, 5-HT modulation can alleviate various TNFα-mediated inflammatory diseases, depressive disorders linked to systemic inflammation and GI disorders. Also, in solid tumor microenvironments, tryptophan-catabolizing enzyme indoleamine 2,3-dioxygenase is present at high concentrations (216, 217). This may impair the synthesis of 5-HT, a tryptophan metabolite, and decrease 5-HT autocrine signaling in T cells (87). Consequently, this may downregulate anti-tumor T cell immunity. Optimization of 5-HT signaling should thus be helpful in cancer.

### Substance P

Substance P (trifluoroacetate salt powder; SP) is an undecapeptide of the tachykinin neuropeptide family. SP is released from the terminals of specific sensory nerves in brain regions of the CNS regulating emotion (hypothalamus, amygdala, and the periaqueductal gray) as well as in most peripheral regions (218, 219). In addition, it is secreted by immune cells such as macrophages, eosinophils, lymphocytes, and dendritic cells. SP exerts its biological activity through G-protein–coupled neurokinin receptors (NKRs), namely, neurokinin 1 receptor (NK1R), NK2R, and NK3R (220). SP has the highest affinity to its endogenous receptor NK1R that is primarily expressed in epithelial cells, fibroblasts and immune cells (218, 219, 221). The NKRs activate phospholipase C, protein kinase C and adenylyl cyclase, and mobilize intracellular Ca^2+^ (219). SP and NK1R are found in close association with serotonin and neurons containing norepinephrine (222). The NKR promoter contains regions that are sensitive to cAMP, AP-1, AP-4, CEBPB, and epidermal growth factor (223). Because these regions are related to cytokine-triggered signal transduction pathways, cytokines are likely to induce NK1R transcription factors (224). NK1R is distributed over cytoplasmic membranes of many cell types, including neurons, glia, endothelia of capillaries, lymphatics, fibroblasts, stem cells, and leukocytes. SP can thus influence or excite many cellular processes (219, 225).

SP acts as an immediate defense, repair and survival system in response to noxious stimuli (stressors) that may compromise biological integrity. Upon tissue damage, infection or other types of stress, SP is released from skin, musculoskeletal, respiratory, or GI tract cells and regulates permeability and contractibility of the organs within those systems (218, 219, 226). SP signaling is known to associate with inflammatory processes, pain perception, modulation of behavior, stress responses, immune function, and vomiting reflex. Furthermore, common among biological processes, SP release and expression of NKRs may not subside in diseases marked by chronic inflammation, such as cancer.

The SP-NK1R system mediates interactions between neurons and immune cells, and modulates several aspects of the immune response, including leukocyte proliferation and activation, and cytokine expression (227). Reciprocally, cytokines may induce expression of SP and NK1R (228–230). Also, SP is involved in neurogenic inflammation, which is a local inflammatory response to certain types of infection or injury (231). Various effects of SP on immune cells are summarized in Table 3.

**Table 3:** Summary of the effects of substance P on immune cell responses.

**Neurotransmitter**	**Receptors**	**Target immune cells**	**Implications in disease**
**Substance P** 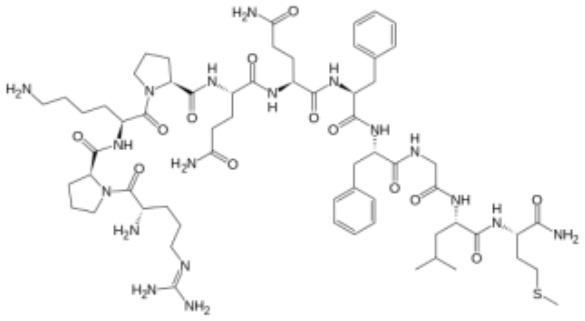	NK1R NK2R NK3R	Neutrophil Macrophage T cell B cell	Asthma Dermatitis IBD Rheumatoid Arthritis Psoriasis Diabetes Cancer
**Cellular adhesion and homing**			
Chemoattracts lymphocytes and upregulates their integrins LFA-1 and VLA-4 to promote migration and extravasation into the target sites (226) NK1R signaling increases the expression of the cellular adhesion molecules ICAM-1 and VCAM-1 on CNS endothelia (219, 226) Reduces CD8^+^T cell migration (119)			
**Lymphocyte function**			
NK1R signaling increases the secretion of pro-inflammatory T_H1_ cytokines IFNγ and TNFα (219, 226) Enhances T cell IL-2 secretion and proliferation (232) Favors T_H17_ differentiation and IFNγ production (218) Enhances B cell Ig secretion (219) Truncated variant of NK1R with a lower affinity to SP prevents lymphocyte overactivation (219) NK1R downstream signaling in NK cells inhibit their degranulation and cytotoxicity by reducing intracellular calcium flux and pERK (233)			
**Myeloid cell function**			
Increases human monocyte secretion of IL-6, IL-23, and TNF-Like 1A (218) Promotes innate inflammation upon secretion following tissue damage, infection or stress (226)			

*LFA-1, Lymphocyte function-associated antigen-1, α_L_β_2_-integrin; VLA-4, Very late antigen-4, α_4_β_1_-integrin, CD49d/CD29; ICAM-1, Intercellular adhesion molecule-1; VCAM-1, Vascular cell adhesion molecule-1; ERK, Extracellular signal-regulated kinases*.

#### Immunostimulatory Effects

SP can help prolong an immune response critical for host survival. SP secretions are known to be increased during recruitment of innate immune cells to target sites causing an amplified immune inflammation by increasing cytokine production (234). SP levels are elevated in the immediate vicinity of lymphocytes (119). In a mouse model of EAE, NK1R antagonist reduced expression of the cellular adhesion molecules ICAM-1 and VCAM-1 on CNS endothelia and the secretion of pro-inflammatory T_H1_ cytokines IFNγ and TNFα (219, 226). SP acts as a chemoattractant for lymphocytes and upregulates their integrins LFA-1 and VLA-4 to promote migration and extravasation into the target sites (226). SP induces IFNγ secretion in human T cells and promotes T_H1_ response (218, 226). This may be facilitated through SP enhanced DC-T cell interaction as blockade of NK1R interaction by a specific antagonist or use of NK1R knockout mice led to reduced T cell proliferation (232). In the absence of DC, proliferation of T cells was partly dependent on signaling through NK1R, revealing an autocrine effect of SP production by T cells. In addition, SP-activated human monocytes secrete IL-6, IL-23, and TNF-Like 1A (TLA-1), promoting T_H17_ development. More specifically, TL1-A engagement with death receptor 3 on activated T cells promote T_H17_ differentiation and IFNγ production (218). Both high TL1A expression and presence of T_H17_ populations during inflammatory and autoimmune diseases have been associated with high expression of SP and its receptor NK1R. Thus, SP may act locally on the myeloid and lymphoid cell interaction to amplify T_H1_ or T_H17_ mediated inflammatory responses.

#### Immunosuppressive Effects

CD8^+^T lymphocytes show a reduced migratory activity in the presence of SP (119). Moreover, NKRs on the surface of immune cells can be endocytosed and recycled to initiate the resolution of inflammation or prevent constitutive activation of immune cells. When endocytosed, NKRs form a complex with β-arrestin and Src that subsequently activates ERK1/2 and mediates a balance between proliferative and anti-apoptotic effects observed in rodent cell lines (235). A truncated variant of NK1R that has a lower affinity to SP can prevent overactivation of immune cells (219). Human NK cells express truncated and full-length NK1R isoforms. NK1R signaling modulated NK cell activation and inhibited NK cell degranulation and cytotoxicity by reducing intracellular calcium flux and pERK (233). This may contribute to the impairment of NK cell function in certain disease states associated with increased circulating SP.

#### Clinical Implications

In diseases associated with chronic inflammation, antagonism of the SP-NK1R system has been studied as a therapeutic intervention (219, 236). SP has a major impact on the pathology of fatal asthma. Asthma patients typically have increased SP-secreting non-adrenergic non-cholinergic (NANC) nerves and express higher levels of NK1R within the respiratory tract, which results in bronchoconstriction, mucus accumulation, vasodilation, and alveolus leakage (219, 237). SP promotes neutrophil chemotaxis to the lung by upregulating the expression of integrins for extravasation, chemokine receptors, degranulation, respiratory burst, IL-1β, and TNFα secretion (219, 238, 239). Another contributing factor is the inability of most asthma patients to produce sufficient amounts of neutral endopeptidase (NEP) to metabolize SP to an inactive compound (237). Thus, to attenuate immune cell recruitment and inflammation of the respiratory tract, NK1R antagonists such as CP96345 serve as potential therapeutic agents besides corticosteroid administration (226). Other contributors to SP-associated respiratory disease involve persistent infection by pathogens such as respiratory syncytial virus (RSV) and cigarette smoke. While virus infection results in constant inflammation attempting to trigger pathogen clearance, cigarette smoke induces NANC nerves to release SP and downregulate airway neutral endopeptidase activity (240, 241).

Similarly, in IBD SP-secreting NANC nerves in the enteric nervous system regulate GI contractility, permeability, and immune responsiveness. Unlike IBD, dermatitis involves inflammation due to physical stress such as scratches or lesions on the skin that result in SP release from NANC within the dermis and facilitate leukocyte recruitment to the target site causing swelling, itching, and redness. This effect is more pronounced in atopic dermatitis patients, for they generally have increased SP-secreting nerves and, thus, a higher concentration of SP in the skin (242, 243). In psoriasis patients also, SP is increased in psoriatic plaques and is responsible for IL-32 synthesis in keratinocytes, which in turn release pro-inflammatory cytokines and activate mast cells. The increased presence of inflammatory mediators and the concentration of SP may predict the severity of psoriasis (244). Likewise, arthritis exhibits a dense innervation of SP-secreting nerves in the joints with synoviocytes expressing abnormally high levels of NK1R. Accordingly, increased SP in the synovial fluid can lead to persistent inflammation of joints (219, 245, 246). In Type I and II diabetes models, treatment with SP reduced pancreatic β-cell destruction by decreasing activation of pancreatic stellar cells, which are responsible for β-cell apoptosis (247). In cancer patients, treatment with NK1R antagonist, aprepitant, is used to prevent the nausea and vomiting that accompany chemotherapy (248).

### Glutamate

L-glutamate, an anion of glutamic acid (2-aminopentanedioic acid; Glu), is one of the most abundant excitatory neurotransmitter present in every type of animal that has a nervous system, including the lower marine invertebrate ctenophores (249). It accounts for over 90% of the synaptic connections in vertebrates (250). It is synthesized from glutamine in the CNS by the enzyme glutaminase, and from α-ketoglutaric acid produced in the citric acid cycle. Because of its role in synaptic plasticity, Glu mediates cognitive processes of learning and memory formation in the CNS (251). In the peripheral nervous system (PNS), Glu regulates nociception as well as cardiovascular, respiratory, reproductive, and immune functions (104).

Glu is present in nearly all organs and tissues of the body as a non-essential amino acid. This made its function as a neurotransmitter difficult to be accepted until injections of Glu into the cerebral ventricles of dogs caused them to have seizures (252). It is released from synapses at high concentrations (10^−3^ M) or circulate in the blood plasma at lower concentrations (10^−5^-10^−4^ M) (253). Since excessive Glu can cause excitotoxicity that induces cell death, it is tightly regulated in the brain as well as in the periphery. It is actively transported out of the nervous system by a high affinity transport system, which maintains its concentration in brain fluids constant (254).

There are two receptor families for Glu: Metabotropic glutamate receptors (mGluRs) and ionotropic glutamate receptors (iGluRs). The metabotropic family includes GPCRs acting through second messenger systems to create slow sustained effects on their targets. There are eight mGluRs divided into three subgroups. Group I receptors (mGluR_1_ and mGluR_5_) are coupled to G_q_ that increases IP3 and diacyl glycerol by activating phospholipase C, and group II (mGluR_2_ and mGluR_3_) and III (mGluR_4_, mGluR_6_, mGluR_7_, and mGluR_8_) are coupled to G_i/o_ that decreases cAMP levels by inhibiting adenylyl cyclase (255). Both CNS and PNS use mGluRs (253).

The ionotropic receptors comprise three families that open membrane channels to allow ions to pass through when cells are activated. There are three types of iGluRs: α-amino-3-hydroxy-5-methyl-4-isoxazolepropionic acid receptors (AMPAR), N-methyl-D-aspartate receptors (NMDAR), and kainate receptors (KARs). AMPARs are most abundant and ubiquitously expressed and are specialized for fast excitation. They increase membrane permeability for Na^+^ and K^+^ and can produce excitatory electrical pulses in the synapses in a fraction of a millisecond after being stimulated. AMPAR are comprised of homo- or hetero-tetramers of GluA1, GluA2, GluA3, and GluA4 subunits. Glu binds to two or more of the AMPAR subunits that allow the ion channel to open for Ca^2+^/Na^+^ influxes or K^+^ efflux, except they inhibit Ca^2+^ influx if GluA2 is involved (253). NMDARs are voltage-gated receptors that increase membrane permeability for Ca^2+^ when activated. They are predominantly expressed in the CNS for learning and memory. They exist as hetero-oligomers comprised of two GluN1 subunits and a combination of GluN2A, GluN2B, GluN2C, GluN2D, GluN3A, and GluN3B, which serve as regulatory subunits to form a tetramer or pentamer. Glycine is a co-activator for NMDAR to open ion channels for cellular Ca^2+^/Na^+^ influx and K^+^ efflux. Glu has higher affinity to AMPAR than to NMDAR (253). KARs are homo or hetero tetramers comprised of GluK1, GluK2, GluK3, GluK4, and GluK5 subunits that, like AMPARs, also facilitate Na^+^ influx and K^+^ efflux. KARs are restricted to the brain and their functions are largely unknown.

**Figure 4:**
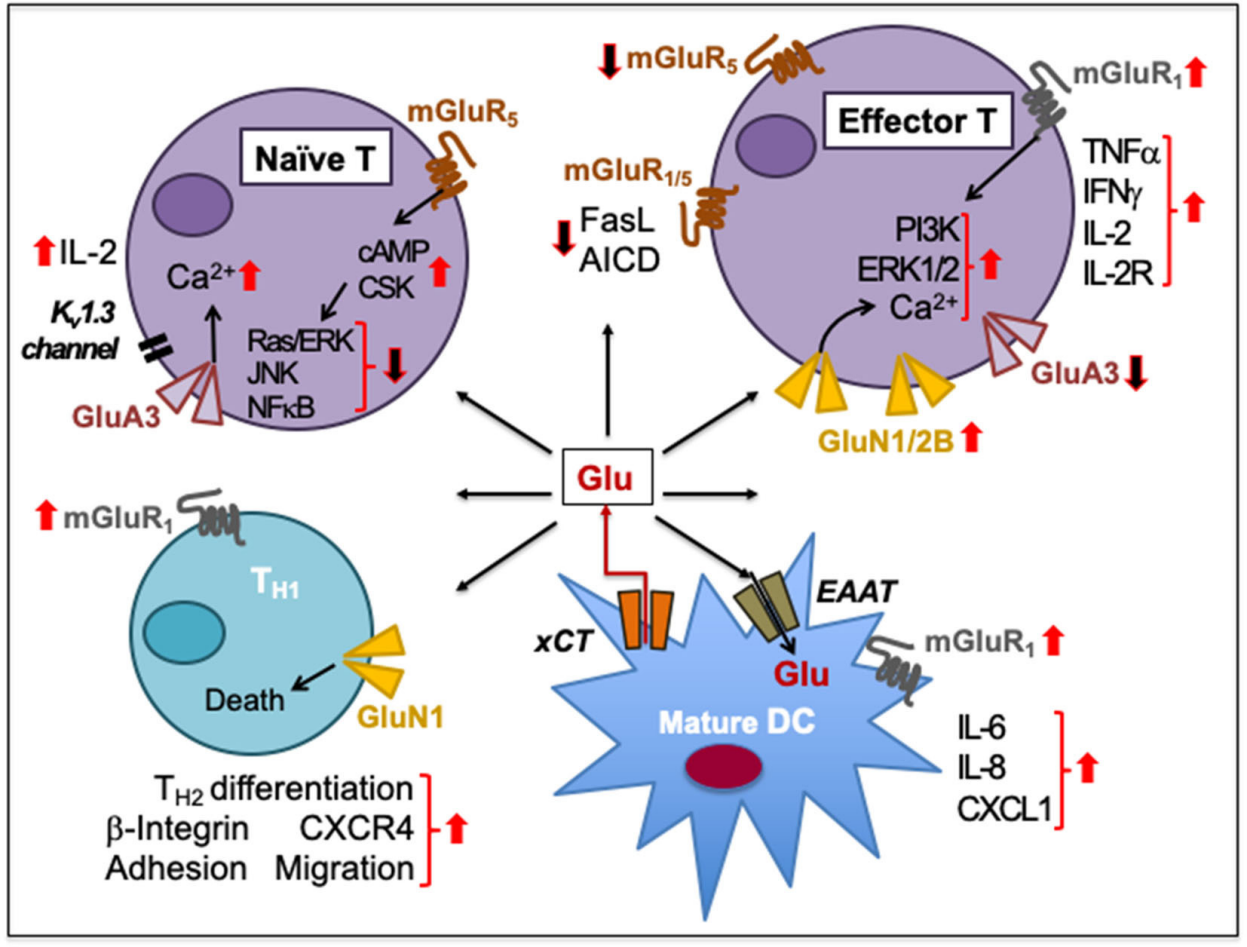
Glutamate-mediated cell-to-cell communication among immune cells. Naïve human peripheral blood T cells express GluA3 and mGluR_5_. Signaling through GluA3 increases Ca^2+^ influx, IL-2 secretion through negative membrane potential of a voltage-gated K^+^ channel K_v_1.3, and IL-2 receptor upregulation during T cell activation. The activation is resisted by the increased expression of mGluR_5_ on naïve T cells and by increased cAMP levels that inhibit Ras/ERK, JNKs, and NFκB and activate C-terminal Src kinase (CSK). Upon activation, T cells express GluN1/2B to sustain increased Ca^2+^ influx. The upregulation of GluN1/2B may downregulate GluA3, which is cleaved through granzyme B. Activation of mGluR_1/5_ on effector T cells prevents the activation-induced cell death (AICD) by decreasing FasL expression. In activated T cells, mGluR_1_ signaling increases PI3K and ERK1/2 signaling. mGluR1 activation also increases IFNγ, TNFα, IL-2, and IL-2R expression during T cell proliferation and effector differentiation, possibly through Glu released from mature DCs through their plasma membrane-expressed cystine-glutamate antiporter (xCT). DCs also express transmembrane excitatory amino acid transporters (EAAT) to regulate extracellular concentrations of Glu. Activation of mGluR1 on DCs facilitates the release of IL-6, IL-8, and CXCL1 production. Activation of both GluN1 and mGluR1 on CD4^+^T_H_ cells induces β-integrin-mediated adhesion to fibronectin and laminin as well as facilitates CXCR4-mediated chemotactic migration. GluN1 signaling causes T_H1_ cell death but supports T_H2_ responses. Upward solid red arrows depict upregulation of indicated molecules or effects whereas the downward solid arrows indicate the reverse. Thin red line arrows indicate cells that produce Glu.

The expression of GluRs and Glu transporters in lymphoid and myeloid cells, as well as the concentration of Glu in which immune cells are exposed to, can induce a pro or anti-inflammatory response (253, 256–259). While GluRs are expressed mostly on lymphoid cells, Glu transporters are prevalent in antigen presenting cells such as DC and macrophages. Various effects of Glu signaling in immune cells are summarized in Table 4 with Glu-mediated immune cell-to-cell communication depicted in Figure 4.

**Table 4:** Summary of the effects of glutamate on immune cell responses.

**Neurotransmitter**	**Receptors**	**Target immune cells**	**Implications in disease**
**Glutamate** 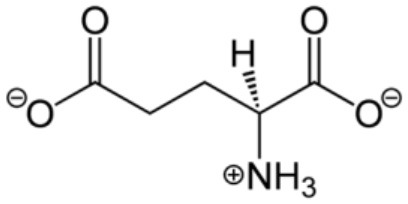	AMPAR (GluA1-4) NMDAR (GluN1, GluN2A-D, GluN3A-B) KAR (GluK1-5) mGluR_1−8_	Neutrophil Macrophage DC T cell B cell	Seizures Epilepsy Encephalomyelitis Multiple sclerosis Cancer
**Cellular adhesion and homing**			
AMPAR signaling induces β-integrin-mediated T cell adhesion to fibronectin and laminin (88) AMPAR signaling increases the CXCR4-mediated T cell chemotactic migration toward chemokine CXCL12 (88) mGluR_1_ signaling induces neutrophil transmigration (260)			
**Lymphocyte function**			
Naïve T cells constitutively express GluA3 and mGluR_5_ receptors (88, 261) Activated T cells express mGluR_1/5_ and NMDAR GluN1 and/or GluN2B subunits (253) NMDARs are upregulated on activated T cells to compensate for GluA3 downregulation due to autocrine/paracrine granzyme B-mediated proteolytic cleavage (262) GluN1 is upregulated on CD4^+^T cells, and leads to T_H1_ cell death and prevalence of T_H2_ responses (263) iGluR mediates Ca^2+^ influx through a voltage-gated K^+^ channel K_v_1.3 (264), and increases IL-2 secretion and IL-2 receptor in T cells (265, 266) mGluR activation increases Ca^2+^ influx in T cells (266, 267) mGluR_1/5_ signaling prevents the activation-induced cell death of activated T cells by decreasing FasL expression (268, 269) mGluR_1_ signaling increases PI3K-Akt and ERK1/2 signaling, and promotes T cell activation, proliferation and secretion of T_H1_ cytokines IFN-γ, TNFα, IL-2, and IL-6 (261) mGluR_5_ signaling inhibits T cell activation and proliferation through increased cAMP levels that suppress the activation of Ras/ERK, JNK, and NFκB but activates C-terminal Src kinase (270) Glu production from glutamine hydrolysis produces epigenetic effects on chromatin accessibility and gene expression to promote T_H17_ but constrain T_H1_ and effector T cell differentiation (271) Proteolytic breakdown of GluA3 into circulation facilitates the production of autoantibodies (262, 272)			
**Dendritic cell maturation and myeloid cell function**			
Mature DCs release Glu through their plasma membrane-expressed Xc-cystine-glutamate antiporter (273) DCs express transmembrane excitatory amino acid transporters that regulate Glu extracellular concentrations (270, 274) mGluR_1_ signaling upregulates myeloid cell production of inflammatory chemoattractants such as CXCL1, IL-6, and IL-8 (260)			

*Glu, Glutamate; AMPAR, α-amino-3-hydroxy-5-methyl-4-isoxazolepropionic acid receptors; NMDAR, N-methyl-D-aspartate receptors; KAR, kainate receptors; iGluR, Ionotropic glutamate receptors; mGluR, Metabotropic glutamate receptors; PI3K, Phosphatidylinositol 3-kinase; Akt, Protein kinase B*.

#### Immunostimulatory Effects

Activation of AMPAR induces β-integrin-mediated T cell adhesion to fibronectin and laminin. It also increases the CXCR4-mediated peripheral T cell chemotactic migration toward the chemokine CXCL12 (88). Stimulation of iGluRs during human T cell activation increases Ca^2+^ influx, IL-2 secretion and IL-2 receptor upregulation (265, 266). The iGluR-mediated Ca^2+^ influx requires negative membrane potential through a voltage-gated K^+^ channel K_v_1.3 that is activated by Glu (264). The activation of mGluRs also increases Ca^2+^ influx (266, 267). Group I mGluRs prevent the activation-induced cell death by decreasing FasL expression that is normally expressed on activated T cells (268, 269). Thus, while iGluRs appear to support early events of TCR signaling through Glu-activated K_v_1.3 K^+^ channels, mGluR signaling sustains T cell activation and survival.

Naïve T cells constitutively express GluA3 and mGluR_5_ (88, 261). In contrast, activated human peripheral blood T cells express mGluR_1/5_ as well as GluN1 and/or GluN2B subunits (253). GluN1 is upregulated upon activation in human T cells to increase Ca^2+^ influx (275). NMDARs are upregulated on activated T cells most likely to compensate for GluA3 downregulation due to autocrine/paracrine granzyme B-mediated proteolytic cleavage (262). Furthermore, mature human DCs release significant amounts of Glu through their plasma membrane-expressed cystine-glutamate antiporter (xCT) (273). DCs also express transmembrane excitatory amino acid transporters (EAAT) that regulate extracellular concentrations of Glu to manage its excitotoxicity (270, 274). EAATs also function as antiporters on an electrochemical gradient of Na^+^, K^+^, and H^+^ concentrations, whereby one molecule of Glu is imported along with three Na^+^ and one H^+^ in exchange of one K^+^ export. In the presence of DC's secreted Glu, while T cell activation is inhibited by an increased expression of mGluR_5_ (270), upregulation of mGluR_1_ in activated T cells counteracts its function (261). After productive Ag presentation by DC, mGluR_1_ signaling in T cells enhances their proliferation and secretion of T_H1_ pro-inflammatory cytokines. Depletion of Glu or mGluR_1_ blockade by antagonist CPCCOEt decreases human T cell proliferation and T_H1_ differentiation, and reduces IFN-γ, TNFα, IL-2, and IL-6 secretion (273). Moreover, activated T cells need to resist quiescence to develop and sustain effector functions. Glu plays a critical metabolic role to meet the energy demands of this activity. In addition, autocrine Glu impacts initial events during GluR-TCR interaction (MTP de Aquino, Hodo et al., manuscript in preparation). Activated T cells upregulate amino acid transporters (276) and enzymes that metabolize glutamine (277). Glu production upon glutamine hydrolysis by the enzyme glutaminase (278) produces epigenetic effects on chromatin accessibility and gene expression that promote T_H17_ but constrain T_H1_ and effector T cell differentiation (271). Accordingly, Glu can impact T cell function at multiple levels of activation and differentiation.

#### Immunosuppressive Effects

GluR signaling can contribute to immunosuppression. GluN1 is rapidly upregulated on CD4^+^ T cells, and leads to T_H1_ cell death and prevalence of T_H2_ responses in humans, interfering with the resolution chronic inflammatory diseases (263). DC-derived Glu stimulating the constitutively expressed mGluR_5_ on T cells blocks their activation. This is mediated through increased cAMP levels that suppress the activation of Ras/ERK, JNK, and NFκB but activates C-terminal Src kinase (270). Conversely, mGluR_5_ antagonism with 2-methyl-6-(phenylethynyl)pyridine enhances T cell proliferation, suggesting that mGluR_5_ may hinder T cell proliferation (273).

Thus, differential expression of GluRs on T cells could be another layer of regulation wherein upon non-cognate antigen presentation by DC mGluR_5_ signaling blocks T cell activation and mGluR_1_ signaling promotes T cell activation following the cognate antigen–TCR binding. During DC–T cell interaction, Glu and its receptors could be an effective system for regulating T cell-mediated responses. Depending on the T cell state (resting or activated), selective Glu agonists could decide T cell fate by inducing excitotoxic death via ionotropic NMDAR, by stimulating activation and proliferation via mGluR_1_ or inhibiting T cell activation via mGluR_5_ (89, 270, 279).

#### Clinical Implications

The immunomodulatory ability of Glu through GluRs expressed on lymphocytes is of clinical importance in autoimmune inflammatory diseases and cancer. Uncontrolled seizures and epileptic episodes are known to be caused by excessive Glu, activating ionotropic GluA3 and GluN1/GluN2 receptors. Also, the granzyme-B-mediated proteolytic breakdown of T cell GluA3 into circulation may facilitate the production of autoantibodies by recognizing those residues as foreign antigens and contribute to the pathogenesis of autoimmune epilepsy (262, 272). Similarly, GluA3-overexpressing autoreactive T cells could attack the myelin of nerves causing demyelinating diseases such as MS (280). In a mouse model of encephalomyelitis, GluR-expressing T cells were autoreactive to myelin basic protein (88).

GluR signaling can contribute to inflammatory environment wherein cancer cells thrive to sustain their growth and survival. In addition, GluR signaling can promote oncogenic signaling. T-leukemia and T-lymphoma cells overexpress mGluRs that promote intracellular Ca^2+^ and proliferative genes *c-fos/c-jun* (253). AMPA iGluRs increase the expression of matrix metalloprotein-9 (MMP-9) via MMP-inducer CD147 and promote tumor cell metastasis (281). Prostate cancer cells express prostate-specific membrane antigen (PSMA), a Glu carboxypeptidase II, also called folate hydrolase-1, which hydrolyzes Glu from vitamin B9 (folic acid) and other substrates. Glu thus released binds to GPCR mGluR_1_ to activate PI3K-Akt signaling that supports prostate cancer progression. The use of inhibitors against mGluR_1_ and PSMA in preclinical models regressed prostate cancer (282). Similarly, mGluR_1_ signaling in triple negative breast cancers promotes their progression, angiogenesis, and metastasis (260, 283). Also, mGluR_1_ and NMDAR antagonists increased dendritic branching and decreased the motility, migration and proliferation of human melanoma cells in xenograft models by inhibiting dynamin GTPase and blocking ERK1/2 pathway (284–286). Apparently, mGluR_1_ signaling has a direct effect on tumor cell growth and survival but it also increases inflammation within the tumor microenvironment by upregulating the production of inflammatory chemoattractants such as CXCL1, IL-6, and IL-8 and inducing neutrophil transmigration (260). Thus, blocking specific GluR signaling, which is exploited by tumors to survive and grow, and enhance tumor-promoting inflammation, could be a potential therapeutic approach to treat tumors and some autoimmune inflammatory diseases.

## Therapeutic Potential of Neuroimmune Modulation

An extensive crosstalk between immune cells and neurotransmitters has a bearing on human diseases. It is appears that immune effects mediated by DA are first pro-inflammatory T_H1_-type, then switching to anti-inflammatory T_H2_-type following activation (104, 125). A collaborative DA-mediated network of DC, T_H_ and B cells along with decreased T_reg_ suppressive activity could be helpful in sustaining CD8^+0^T cell effector responses in immunosuppressive pathologies. Therefore, by selectively triggering specific DR on immune cells DA may provide for an immunological behavior-switching and context-dependent immune stimulatory or inhibitory responses. This offers DA-mediated treatment alternatives to mitigate neuropathologies and autoimmune diseases. DA agonists may help in brain disorders such as PD whereas specific DR antagonists could help alleviate MS, schizophrenia, AD, colitis, and IBD.

5-HT modulation can alleviate various TNFα-mediated inflammatory diseases, including depressive disorders linked to systemic inflammation, GI disorders, atherosclerosis, rheumatoid arthritis, psoriasis, type II diabetes, schizophrenia, and AD. Activation of 5-HT_2A_ represents a potential therapeutic avenue for the treatment of such disorders. Moreover, the inhibitory effects of 5-HT_2A_ agonists on TNFα-mediated inflammation last many hours after the administration of TNFα (184). Consequently, 5-HT-agonist-based therapies may also treat inflammation-associated tissue stress or damage. In addition, 5-HT may alleviate symptoms associated with asthma, such as severe bronchoconstriction, mucus buildup, and eosinophil-induced inflammation in response to allergens (169, 175). Also, optimization of 5-HT signaling should be helpful in cancer as in tumor microenvironments tryptophan-catabolizing enzyme indoleamine 2,3-dioxygenase is present at high concentrations (216, 217), which may impair the synthesis of 5-HT and decrease 5-HT autocrine signaling in T cells (87). In diseases associated with chronic inflammation, antagonism of the SP-NK1R system is under investigation as a therapeutic intervention (219, 236). In cancer patients, treatment with NK1R antagonists is already used to prevent the nausea and vomiting that accompany chemotherapy (248).

The expression of GluRs and Glu transporters in lymphoid and myeloid cells, as well as the concentration of Glu in which immune cells are exposed to, can induce a pro or anti-inflammatory response (253, 256–259). GluR signaling can impact T cell activation and differentiation at multiple levels. While iGluRs appear to support early events of TCR signaling through Glu-activated K_v_1.3 K^+^ channels, mGluR signaling sustains T cell activation and effector function. Also, differential expression of GluRs on T cells could be another layer of regulation wherein upon non-cognate antigen presentation by DC mGluR_5_ signaling blocks T cell activation and mGluR_1_ signaling promotes T cell activation following the cognate antigen–TCR binding. Selective Glu agonists could affect T cell fate by inducing excitotoxic death via ionotropic NMDAR, by stimulating activation and proliferation via mGluR_1_ or inhibiting T cell activation via mGluR_5_ (89, 270, 279). Moreover, Glu can contribute to inflammatory microenvironment wherein cancer cells thrive to sustain their growth and survival. Blocking specific GluR signaling that is exploited by tumors to enhance tumor-promoting inflammation could be a potential therapeutic approach.

The neuroimmune approaches could be harnessed to improve antitumor responses in metastatic cancers where we are far from achieving the goal of preventive or therapeutic modality assuring relapse-free survival (287). Tumor cells show chemotaxis toward neurotransmitters such as DA and other catecholamines (288). A neuro-neoplastic synapse has been observed following direct interactions of tumor cells with nerve cells (289). Tumors may also initiate their own innervation by the release of neurotrophic factors. Studies have revealed a prominent role of neurotransmitters in the tumor microenvironment contributing to the pathogenesis of cancers (290). Besides the acute “fight or flight” response triggered by catecholamines from sympathetic nerve terminals and adrenal glands, the sympathetic nervous system can regulate gene expression in the tumor microenvironment (291). Although the SNS-mediated gene regulation or molecular mobility and defense programs have been correlated with tumor progression and metastasis (291, 292), they can be manipulated to cause tumor regression (293, 294). SNS ablation in an acute myelogenous leukemia model caused infiltration of leukemic cells into the bone marrow (294), suggesting separate mesenchymal niche activities for malignant and healthy hematopoietic stem cells in the bone marrow.

In head and neck cancer patients, adoptive transfer of peripheral blood T cells treated with Glu and DA promoted their CXCL12-guided migration to autologous tumors and elevated the expression of TCR downstream molecules (295). Another study showed that stimulation of the reward system through dopaminergic neurons in ventral tegmental area decreased tumor growth in mouse models by reducing myeloid-derived suppressor cells and increasing granzyme-B expression in tumor-infiltrating CD8^+^T cells. A strong connection also exists between the mental state of the patient and cancer resolution by the immune system (12).

**Figure 5:**
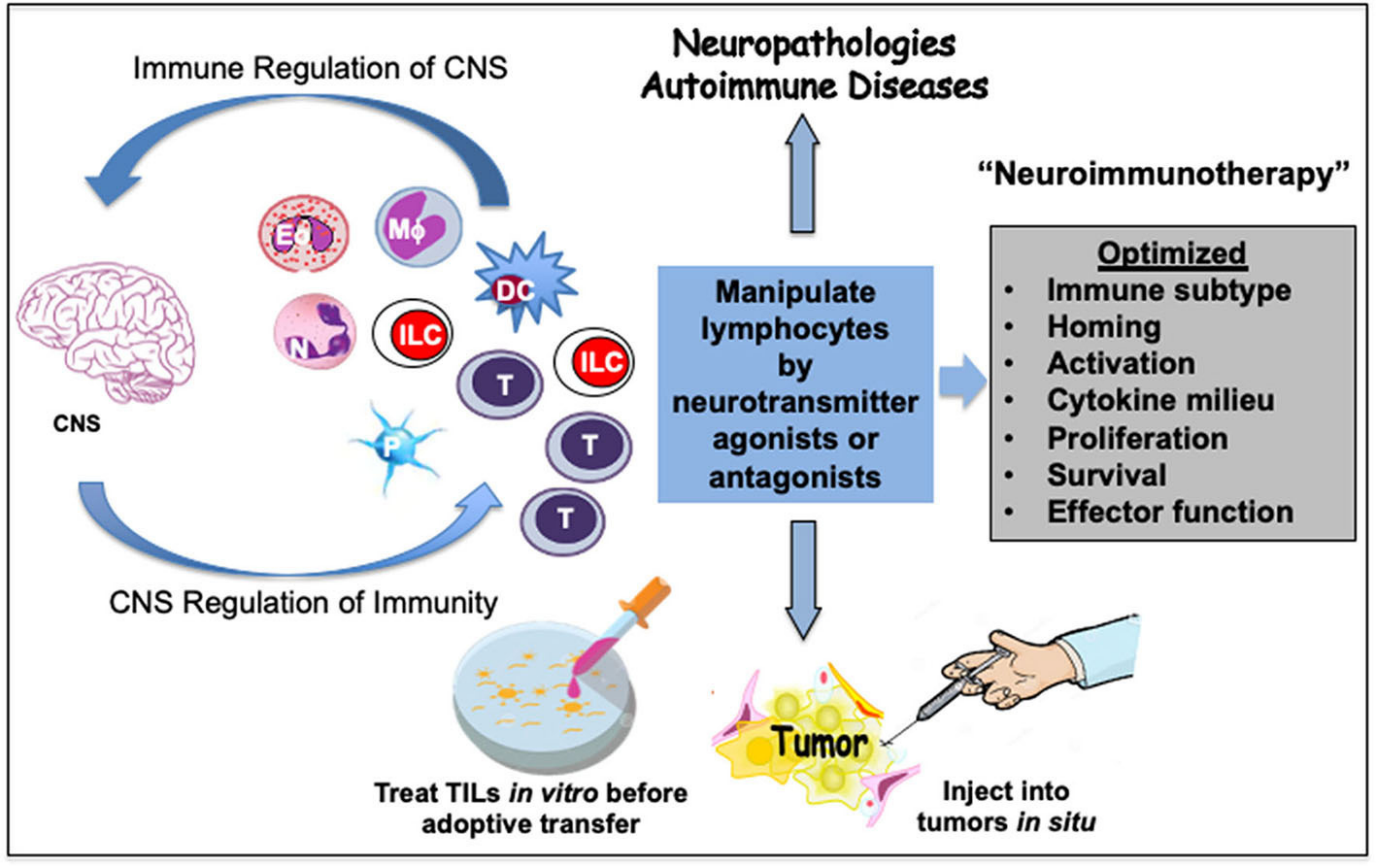
Prospective neuroimmunotherapy scheme to optimize immune function in cancer and autoimmune pathologies. A crosstalk between immune cells and the central nervous system (CNS) has a bearing on human diseases. This offers treatment alternatives to mitigate cancer, neuropathologies and autoimmune diseases. The neurotransmitters present a high potential to act as therapeutic supplements to optimize the function of immune cells including eosinophils (Eo), neutrophils (*N*), macrophages (Mϕ), innate lymphoid cells (ILC), dendritic cells (DC), or T cells. Some immune cells such as platelets (*P*) serve as stores of neurotransmitters. Select neurotransmitter agonists or antagonists could be developed and tested in combination with other therapeutic modalities to suppress or stimulate immune responses, respectively. Such an optimized neuroimmunotherapy could be helpful in debilitating chronic immunopathologies. Activation of neurotransmitter receptors on lymphocytes by select neurotransmitters can modulate their migration and homing as well as differentiation, cytokine secretion, proliferation, survival, or effector function. As an example, adoptive T cell therapy for cancers can be improved by treating harvested tumor-infiltrating lymphocytes (TIL) with select neurotransmitter agonists or antagonists *in vitro* before transfer. Alternatively, neurotransmitter agonists or antagonists can be injected into the solid tumors to manipulate T cells or innate lymphoid cells (ILC) such as NK cells *in situ*. Optimization of such neuroimmunotherapy approaches could provide more effective holistic therapeutic benefits.

The neurotransmitters thus have a high potential to act as therapeutic supplements to boost or optimize immune cell responses. Neurotransmitters or their selective agonists or antagonists could be developed and tested in combination with other therapeutic modalities to suppress or stimulate immune responses, respectively (Figure 5). Such an optimized “neuroimmunotherapy” could be very helpful in debilitating chronic immunopathologies.

## Conclusions and Future Perspectives

Depending on the tissue-specific context, neurotransmitters are either immunostimulatory or immunosuppressive to peripheral immune responses. Various inflammatory pathologies including tumor microenvironment are integrated with the neural, immune, and endocrine systems. DA, 5-HT, SP, and Glu have pro-inflammatory effects on immune cells. On one hand, these pro-inflammatory effects may exacerbate disease severity, on the other, they may be channelized to reverse immunosuppression in chronic diseases. Moreover, neurotransmitter receptors have multiple subtypes that can be selectively triggered to elicit different types of immune responses. A neurotransmitter binding to a receptor on one subset of immune cells can promote an immunostimulatory response while promoting an inhibitory response in another immune subset depending on the repertoire of receptors and signaling targets involved in the downstream cascade of events. For example, in one condition a neurotransmitter may activate migration, cytokine secretion, or integrin expression on naïve T cells while the same neurotransmitter may inhibit these functions when T cells have already been primed or stimulated. The concentration of neurotransmitters in circulation or in the immediate tissue vicinity may also determine the outcome of the elicited immune responses.

From an evolutionary perspective, the concerted activity of the nervous and immune systems is integral to tissue, organ and organismic health. The nervous system senses inflammatory responses and responds by controlling stress-response pathways at the organismal level. G-protein-coupled catecholamine receptors in the primitive nervous system of the nematode *Caenorhabditis elegans* control innate immune responses by regulating unfolded protein response genes in non-neuronal tissues (296). Furthermore, the cellular and molecular signaling axes of neuroimmune interactions can be regulated by circadian rhythms and psychological stress (297, 298). The neuroimmune communication is, thus, an exciting transdisciplinary area of research that is relevant to exploring cures against chronic immunopathologies. Using the advanced technologies of genetic molecular mapping and imaging, high-throughput single-cell analysis and computational systems biology, a comprehensive analysis of neuroimmune modulation and cross systems-network may lead to the discovery of new functional bases and regulatory circuits of neuroimmune networks. Building on these advances, a carefully optimized “neuroimmunotherapy” approach may provide holistic therapeutic benefits in chronic inflammatory diseases without overtly harming the physiological systems of the body.

## Author Contributions

ASha conceived, designed the manuscript, and acquired funding for the work. TH prepared the first draft. MA, AShi, and ASha contributed to thorough review and editing of the manuscript. TH, MA, AShi, and ASha composed tables and figures. All authors read and approved the final manuscript for publication.

## Conflict of Interest

The authors declare that the research was conducted in the absence of any commercial or financial relationships that could be construed as a potential conflict of interest.

## References

[1] Jerne NK. Towards a network theory of the immune system. Ann Immunol. (1974) 125C:373–89. 4142565

[2] Medawar PB Immunity to homologous grafted skin; the fate of skin homografts transplanted to the brain, to subcutaneous tissue, and to the anterior chamber of the eye. Br J Exp Pathol. (1948) 29:58–69. 18865105 PMC2073079

[3] Ransohoff RM Engelhardt B. The anatomical and cellular basis of immune surveillance in the central nervous system. Nat Rev Immunol. (2012) 12:623–35. 10.1038/nri3265 22903150

[4] Kipnis J. Multifaceted interactions between adaptive immunity and the central nervous system. Science. (2016) 353:766–71. 10.1126/science.aag2638 27540163 PMC5590839

[5] Louveau A Plog BA Antila S Alitalo K Nedergaard M Kipnis J. Understanding the functions and relationships of the glymphatic system and meningeal lymphatics. J Clin Invest. (2017) 127:3210–19. 10.1172/JCI90603 28862640 PMC5669566

[6] Korin B Ben-Shaanan TL Schiller M Dubovik T Azulay-Debby H Boshnak NT. High-dimensional, single-cell characterization of the brain's immune compartment. Nat Neurosci. (2017) 20:1300–9. 10.1038/nn.4610 28758994

[7] Korin B Dubovik T Rolls A. Mass cytometry analysis of immune cells in the brain. Nat Protoc. (2018) 13:377–91. 10.1038/nprot.2017.155 29370157

[8] Korin B Rolls A. Application of chemogenetics and optogenetics to dissect brain-immune interactions. Methods Mol Biol. (2018) 1781:195–208. 10.1007/978-1-4939-7828-1_11 29705849

[9] Engelhardt B Molecular mechanisms involved in T cell migration across the blood-brain barrier. J Neural Transm. (2006) 113:477–85. 10.1007/s00702-005-0409-y 16550326

[10] Engelhardt B. Regulation of immune cell entry into the central nervous system. Results Probl Cell Differ. (2006) 43:259–80. 10.1007/400_020 17068976

[11] Ben-Shaanan TL Azulay-Debby H Dubovik T Starosvetsky E Korin B Schiller M. Activation of the reward system boosts innate and adaptive immunity. Nat Med. (2016) 22:940–4. 10.1038/nm.4133 27376577

[12] Ben-Shaanan TL Schiller M Azulay-Debby H Korin B Boshnak N Koren T. Modulation of anti-tumor immunity by the brain's reward system. Nat Commun. (2018) 9:2723. 10.1038/s41467-018-05283-5 30006573 PMC6045610

[13] Pinho-Ribeiro FA Baddal B Haarsma R O'Seaghdha M Yang NJ Blake KJ. Blocking neuronal signaling to immune cells treats streptococcal invasive infection. Cell. (2018) 173:1083–97.e22. 10.1016/j.cell.2018.04.006 29754819 PMC5959783

[14] Shanker A Marincola FM. Cooperativity of adaptive and innate immunity: implications for cancer therapy. Cancer Immunol Immunother. (2011) 60:1061–74. 10.1007/s00262-011-1053-z 21656157 PMC3508514

[15] Shanker Neuroendocrine crosstalk of immunity. J Blood Lymph. (2011) 1:1–2. 10.4172/2165-7831.1000e105

[16] Isern J Garcia-Garcia A Martin AM Arranz L Martin-Perez D Torroja C. The neural crest is a source of mesenchymal stem cells with specialized hematopoietic stem cell niche function. Elife. (2014) 3:e03696. 10.7554/eLife.03696 25255216 PMC4381911

[17] Yamazaki S Ema H Karlsson G Yamaguchi T Miyoshi H Shioda S. Nonmyelinating Schwann cells maintain hematopoietic stem cell hibernation in the bone marrow niche. Cell. (2011) 147:1146–58. 10.1016/j.cell.2011.09.053 22118468

[18] Spiegel A Shivtiel S Kalinkovich A Ludin A Netzer N Goichberg P. Catecholaminergic neurotransmitters regulate migration and repopulation of immature human CD34+ cells through Wnt signaling. Nat Immunol. (2007) 8:1123–31. 10.1038/ni1509 17828268

[19] Katayama Y Battista M Kao WM Hidalgo A Peired AJ Thomas SA. Signals from the sympathetic nervous system regulate hematopoietic stem cell egress from bone marrow. Cell. (2006) 124:407–21. 10.1016/j.cell.2005.10.041 16439213

[20] Ginhoux F Guilliams M. Tissue-resident macrophage ontogeny and homeostasis. Immunity. (2016) 44:439–49. 10.1016/j.immuni.2016.02.024 26982352

[21] Li Q Barres BA. Microglia and macrophages in brain homeostasis and disease. Nat Rev Immunol. (2018) 18:225–42. 10.1038/nri.2017.125 29151590

[22] Gabanyi I Muller PA Feighery L Oliveira TY Costa-Pinto FA Mucida D. Neuro-immune interactions drive tissue programming in intestinal macrophages. Cell. (2016) 164:378–91. 10.1016/j.cell.2015.12.023 26777404 PMC4733406

[23] Zetterstrom BE. Effect of denervation on blood flow in the dog spleen during haemorrhagic shock. Acta Chir Scand. (1973) 139:111–6. 4718162

[24] Greenway CV Stark RD. Vascular responses of the spleen to rapid haemorrhage in the anaesthetized cat. J Physiol. (1969) 204:169–79. 10.1113/jphysiol.1969.sp008906 4310939 PMC1351601

[25] Foster SL Seehus CR Woolf CJ Talbot S. Sense and immunity: context-dependent neuro-immune interplay. Front Immunol. (2017) 8:1463. 10.3389/fimmu.2017.01463 29163530 PMC5675863

[26] Dulken BW Buckley MT Navarro Negredo P Saligrama N Cayrol R Leeman DS. Single-cell analysis reveals T cell infiltration in old neurogenic niches. Nature. (2019) 571:205–10. 10.1038/s41586-019-1362-5 31270459 PMC7111535

[27] Ito M Komai K Mise-Omata S Iizuka-Koga M Noguchi Y Kondo T. Brain regulatory T cells suppress astrogliosis and potentiate neurological recovery. Nature. (2019) 565:246–50. 10.1038/s41586-018-0824-5 30602786

[28] Barnes MA Carson MJ Nair MG. Non-traditional cytokines: how catecholamines and adipokines influence macrophages in immunity, metabolism and the central nervous system. Cytokine. (2015) 72:210–9. 10.1016/j.cyto.2015.01.008 25703786 PMC4590987

[29] Nguyen KD Qiu Y Cui X Goh YP Mwangi J David T. Alternatively activated macrophages produce catecholamines to sustain adaptive thermogenesis. Nature. (2011) 480:104–8. 10.1038/nature10653 22101429 PMC3371761

[30] Besedovsky H del Rey A Sorkin E Dinarello CA. Immunoregulatory feedback between interleukin-1 and glucocorticoid hormones. Science. (1986) 233:652–4. 10.1126/science.3014662 3014662

[31] Turnbull AV Rivier C. Regulation of the HPA axis by cytokines. Brain Behav Immun. (1995) 9:253–75. 10.1006/brbi.1995.1026 8903845

[32] Besedovsky HO A. del Rey: the cytokine-HPA axis feed-back circuit. Z Rheumatol. (2000) 59(Suppl.2):II/26–30. 10.1007/s003930070014 11155800

[33] Blalock JE Smith EM Meyer WJ 3rd. The pituitary-adrenocortical axis and the immune system. Clin Endocrinol Metab. (1985) 14:1021–38. 10.1016/S0300-595X(85)80087-6 3002675

[34] Shanker. Is thymus redundant after adulthood? Immunol Lett. (2004) 91:79–86. 10.1016/j.imlet.2003.12.012 15019273

[35] Savino W Arzt E Dardenne M. Immunoneuroendocrine connectivity: the paradigm of the thymus-hypothalamus/pituitary axis. Neuroimmunomodulation. (1999) 6:126–36. 10.1159/000026372 9876243

[36] Berczi I. The stress concept and neuroimmunoregulation in modern biology. Ann N Y Acad Sci. (1998) 851:3–12. 10.1111/j.1749-6632.1998.tb08969.x 9668600

[37] de Felice C Toti P Musaro M Peruzzi L Paffetti P Pasqui L Magaldi R. Early activation of the hypothalamic-pituitary-adrenal-axis in very-low-birth-weight infants with small thymus at birth. J Matern Fetal Neonatal Med. (2008) 21:251–4. 10.1080/14767050801927871 18330821

[38] Dominguez-Gerpe L. Stress-induced alterations in the programmed natural cycles of post-natal lymphoid organ development in C57BL/6 mice: evidence for a regulatory feedback relationship between bone marrow and thymus. Immunobiology. (2007) 212:613–27. 10.1016/j.imbio.2007.04.005 17869639

[39] Moleriu RD Zaharie D Moatar-Moleriu LC Gruia AT Mic AA Mic FA. Insights into the mechanisms of thymus involution and regeneration by modeling the glucocorticoid-induced perturbation of thymocyte populations dynamics. J Theor Biol. (2014) 348:80–99. 10.1016/j.jtbi.2014.01.020 24486233

[40] Shanker A Singh SM Sodhi A. Ascitic growth of a spontaneous transplantable T cell lymphoma induces thymic involution. 2. Induction of apoptosis in thymocytes. Tumour Biol. (2000) 21:315–27. 10.1159/000030137 11006572

[41] Shanker A Singh SM Sodhi A Ascitic growth of a spontaneous transplantable T cell lymphoma induces thymic involution. 1. Alterations in the CD4/CD8 distribution in thymocytes. Tumour Biol. (2000) 21:288–98. 10.1159/000030134 10940825

[42] Roy R Singh SM Shanker A Sodhi A. Mechanism of thymocyte apoptosis induced by serum of tumor-bearing host: the molecular events involved and their inhibition by thymosin alpha-1. Int J Immunopharmacol. (2000) 22:309–21. 10.1016/S0192-0561(99)00087-9 10689104

[43] Shanker A Singh SM. Immunopotentiation in mice bearing a spontaneous transplantable T-cell lymphoma: role of thymic extract. Neoplasma. (2003) 50:272–9. 12937840

[44] Andersson U Tracey KJ. Neural reflexes in inflammation and immunity. J Exp Med. (2012) 209:1057–68. 10.1084/jem.20120571 22665702 PMC3371736

[45] Martinez FO Sica A Mantovani A Locati M. Macrophage activation and polarization. Front Biosci. (2008) 13:453–61. 10.2741/2692 17981560

[46] Kershaw SG Della Vedova CB Majumder I Ward MB Farquharson AL Williamson PA. Acute opioid administration induces hypothalamic-pituitary-adrenal activation and is mediated by genetic variation in interleukin (Il)1B. Pharmacol Biochem Behav. (2015) 138:9–13. 10.1016/j.pbb.2015.09.005 26363312

[47] Abderrazak A Syrovets T Couchie D El Hadri K Friguet B Simmet T. NLRP3 inflammasome: from a danger signal sensor to a regulatory node of oxidative stress and inflammatory diseases. Redox Biol. (2015) 4:296–307. 10.1016/j.redox.2015.01.008 25625584 PMC4315937

[48] Cavalcanti DM Lotufo CM Borelli P Ferreira ZS Markus RP Farsky SH. Endogenous glucocorticoids control neutrophil mobilization from bone marrow to blood and tissues in non-inflammatory conditions. Br J Pharmacol. (2007) 152:1291–300. 10.1038/sj.bjp.0707512 17982481 PMC2189989

[49] Dubois CM Neta R Keller JR Jacobsen SE Oppenheim JJ Ruscetti F. Hematopoietic growth factors and glucocorticoids synergize to mimic the effects of IL-1 on granulocyte differentiation and IL-1 receptor induction on bone marrow cells *in vivo*. Exp Hematol. (1993) 21:303–10. 7678814

[50] Elsas PX Neto HA Cheraim AB Magalhaes ES Accioly MT Carvalho VF. Gaspar Elsas: induction of bone-marrow eosinophilia in mice submitted to surgery is dependent on stress-induced secretion of glucocorticoids. Br J Pharmacol. (2004) 143:541–8. 10.1038/sj.bjp.0705943 15381631 PMC1575426

[51] Roggero E Perez A Tamae-Kakazu M Piazzon I Nepomnaschy I Besedovsky HO. Endogenous glucocorticoids cause thymus atrophy but are protective during acute *Trypanosoma cruzi* infection. J Endocrinol. (2006) 190:495–503. 10.1677/joe.1.06642 16899582

[52] Wang L Fan J Lin YS Guo YS Gao B Shi QY. Glucocorticoids induce autophagy in rat bone marrow mesenchymal stem cells. Mol Med Rep. (2015) 11:2711–6. 10.3892/mmr.2014.3099 25515523

[53] Kennedy AD DeLeo FR. Neutrophil apoptosis and the resolution of infection. Immunol Res. (2009) 43:25–61. 10.1007/s12026-008-8049-6 19066741

[54] Nathan C Ding A. Nonresolving inflammation. Cell. (2010) 140:871–82. 10.1016/j.cell.2010.02.029 20303877

[55] Maier SF Wiertelak EP Martin D Watkins LR. Interleukin-1 mediates the behavioral hyperalgesia produced by lithium chloride and endotoxin. Brain Res. (1993) 623:321–4. 10.1016/0006-8993(93)91446-Y 8221116

[56] Hoffman HH Schnitzlein HN. The numbers of nerve fibers in the vagus nerve of man. Anat Rec. (1961) 139:429–35. 10.1002/ar.1091390312 13963923

[57] Pochet R Delespesse G Gausset PW Collet H. Distribution of beta-adrenergic receptors on human lymphocyte subpopulations. Clin Exp Immunol. (1979) 38:578–84. 43789 PMC1537922

[58] Reardon C. Neuro-immune interactions in the cholinergic anti-inflammatory reflex. Immunol Lett. (2016) 178:92–6. 10.1016/j.imlet.2016.08.006 27542331

[59] Guyot M Simon T Panzolini C Ceppo F Daoudlarian D Murris E. Apical splenic nerve electrical stimulation discloses an anti-inflammatory pathway relying on adrenergic and nicotinic receptors in myeloid cells. Brain Behav Immun. (2019) 80:238–46. 10.1016/j.bbi.2019.03.015 30885844

[60] Borovikova LV Ivanova S Zhang M Yang H Botchkina GI Watkins LR. Vagus nerve stimulation attenuates the systemic inflammatory response to endotoxin. Nature. (2000) 405:458–62. 10.1038/35013070 10839541

[61] Tracey J. Physiology and immunology of the cholinergic antiinflammatory pathway. J Clin Invest. (2007) 117:289–96. 10.1172/JCI30555 17273548 PMC1783813

[62] Wang H Liao H Ochani M Justiniani M Lin X Yang L. Cholinergic agonists inhibit HMGB1 release and improve survival in experimental sepsis. Nat Med. (2004) 10:1216–21. 10.1038/nm1124 15502843

[63] Wang H Yu M Ochani M Amella CA Tanovic M Susarla S. Nicotinic acetylcholine receptor alpha7 subunit is an essential regulator of inflammation. Nature. (2003) 421:384–8. 10.1038/nature01339 12508119

[64] Rosas-Ballina M Olofsson PS Ochani M Valdes-Ferrer SI Levine YA Reardon C. Acetylcholine-synthesizing T cells relay neural signals in a vagus nerve circuit. Science. (2011) 334:98–101. 10.1126/science.1209985 21921156 PMC4548937

[65] Chavan SS Pavlov VA Tracey KJ. Mechanisms and therapeutic relevance of neuro-immune communication. Immunity. (2017) 46:927–42. 10.1016/j.immuni.2017.06.008 28636960 PMC5578398

[66] Huston JM Rosas-Ballina M Xue X Dowling O Ochani K Ochani M. Cholinergic neural signals to the spleen down-regulate leukocyte trafficking via CD11b. J Immunol. (2009) 183:552–9. 10.4049/jimmunol.0802684 19542466 PMC2806576

[67] Wu H Li L Su X. Vagus nerve through alpha7 nAChR modulates lung infection and inflammation: models, cells, and signals. Biomed Res Int. (2014) 2014:283525. 10.1155/2014/283525 25136575 PMC4127262

[68] Zanos TP Silverman HA Levy T Tsaava T Battinelli E Lorraine PW. Identification of cytokine-specific sensory neural signals by decoding murine vagus nerve activity. Proc Natl Acad Sci USA. (2018) 115:E4843–52. 10.1073/pnas.1719083115 29735654 PMC6003492

[69] Goehler LE Relton JK Dripps D Kiechle R Tartaglia N Maier SF. Vagal paraganglia bind biotinylated interleukin-1 receptor antagonist: a possible mechanism for immune-to-brain communication. Brain Res Bull. (1997) 43:357–64. 10.1016/S0361-9230(97)00020-8 9227848

[70] Shu HF Wang BR Wang SR Yao W Huang HP Zhou Z. IL-1beta inhibits IK and increases [Ca2+]i in the carotid body glomus cells and increases carotid sinus nerve firings in the rat. Eur J Neurosci. (2007) 25:3638–47. 10.1111/j.1460-9568.2007.05586.x 17610583

[71] Miller AH Raison CL. The role of inflammation in depression: from evolutionary imperative to modern treatment target. Nat Rev Immunol. (2016) 16:22–34. 10.1038/nri.2015.5 26711676 PMC5542678

[72] Derecki NC Cardani AN Yang CH Quinnies KM Crihfield A Lynch KR. Regulation of learning and memory by meningeal immunity: a key role for IL-4. J Exp Med. (2010) 207:1067–80. 10.1084/jem.20091419 20439540 PMC2867291

[73] Filiano AJ Xu Y Tustison NJ Marsh RL Baker W Smirnov I. Unexpected role of interferon-gamma in regulating neuronal connectivity and social behaviour. Nature. (2016) 535:425–9. 10.1038/nature18626 27409813 PMC4961620

[74] Baruch K Deczkowska A David E Castellano JM Miller O Kertser A. Aging. Aging-induced type I interferon response at the choroid plexus negatively affects brain function. Science. (2014) 346:89–93. 10.1126/science.1252945 25147279 PMC4869326

[75] Baruch K Schwartz M. CNS-specific T cells shape brain function via the choroid plexus. Brain Behav Immun. (2013) 34:11–6. 10.1016/j.bbi.2013.04.002 23597431

[76] Baruch K Ron-Harel N Gal H Deczkowska A Shifrut E Ndifon W. CNS-specific immunity at the choroid plexus shifts toward destructive Th2 inflammation in brain aging. Proc Natl Acad Sci USA. (2013) 110:2264–9. 10.1073/pnas.1211270110 23335631 PMC3568380

[77] Choi GB Yim YS Wong H Kim S Kim H Kim SV. The maternal interleukin-17a pathway in mice promotes autism-like phenotypes in offspring. Science. (2016) 351:933–9. 10.1126/science.aad0314 26822608 PMC4782964

[78] Kostanyan IA Merkulova MI Navolotskaya EV Nurieva RI. Study of interaction between L-glutamate and human blood lymphocytes. Immunol Lett. (1997) 58:177–80. 10.1016/S0165-2478(97)00086-2 9293400

[79] Sato KZ Fujii T Watanabe Y Yamada S Ando T Kazuko F. Diversity of mRNA expression for muscarinic acetylcholine receptor subtypes and neuronal nicotinic acetylcholine receptor subunits in human mononuclear leukocytes and leukemic cell lines. Neurosci Lett. (1999) 266:17–20. 10.1016/S0304-3940(99)00259-1 10336173

[80] Petitto JM Huang Z McCarthy DB. Molecular cloning of NPY-Y1 receptor cDNA from rat splenic lymphocytes: evidence of low levels of mRNA expression and [125I]NPY binding sites. J Neuroimmunol. (1994) 54:81–6. 10.1016/0165-5728(94)90234-8 7929806

[81] Marino F Cosentino M. Adrenergic modulation of immune cells: an update. Amino Acids. (2013) 45:55–71. 10.1007/s00726-011-1186-6 22160285

[82] Kioussis D Pachnis V. Immune and nervous systems: more than just a superficial similarity? Immunity. (2009) 31:705–10. 10.1016/j.immuni.2009.09.009 19836266

[83] Park CK Xu ZZ Berta T Han Q Chen G Liu XJ. Extracellular microRNAs activate nociceptor neurons to elicit pain via TLR7 and TRPA1. Neuron. (2014) 82:47–54. 10.1016/j.neuron.2014.02.011 24698267 PMC3982230

[84] Steinberg BE Silverman HA Robbiati S Gunasekaran MK Tsaava T Battinelli E. Cytokine-specific neurograms in the sensory vagus nerve. Bioelectron Med. (2016) 3:7–17. 10.15424/bioelectronmed.2016.00007 30003120 PMC6039192

[85] Yarlagadda A Alfson E Clayton AH. The blood brain barrier and the role of cytokines in neuropsychiatry. Psychiatry. (2009) 6:18–22. 20049146 PMC2801483

[86] Crack PJ Bray PJ. Toll-like receptors in the brain and their potential roles in neuropathology. Immunol Cell Biol. (2007) 85:476–80. 10.1038/sj.icb.7100103 17667932

[87] Leon-Ponte M Ahern GP O'Connell PJ. Serotonin provides an accessory signal to enhance T-cell activation by signaling through the 5-HT7 receptor. Blood. (2007) 109:3139–46. 10.1182/blood-2006-10-052787 17158224 PMC1852236

[88] Ganor Y Besser M Ben-Zakay N Unger T Levite M. Human T cells express a functional ionotropic glutamate receptor GluR3, and glutamate by itself triggers integrin-mediated adhesion to laminin and fibronectin and chemotactic migration. J Immunol. (2003) 170:4362–72. 10.4049/jimmunol.170.8.4362 12682273

[89] Franco R Pacheco R Lluis C Ahern GP O'Connell PJ. The emergence of neurotransmitters as immune modulators. Trends Immunol. (2007) 28:400–7. 10.1016/j.it.2007.07.005 17689291

[90] Prud'homme GJ Glinka Y Wang Q. Immunological GABAergic interactions and therapeutic applications in autoimmune diseases. Autoimmun Rev. (2015) 14:1048–56. 10.1016/j.autrev.2015.07.011 26226414

[91] Pavlov VA Chavan SS Tracey KJ. Molecular and functional neuroscience in immunity. Annu Rev Immunol. (2018) 36:783–812. 10.1146/annurev-immunol-042617-053158 29677475 PMC6057146

[92] Yoo BB Mazmanian SK. The enteric network: interactions between the immune and nervous systems of the gut. Immunity. (2017) 46:910–26. 10.1016/j.immuni.2017.05.011 28636959 PMC5551410

[93] Veiga-Fernandes H Mucida D. Neuro-immune interactions at barrier surfaces. Cell. (2016) 165:801–11. 10.1016/j.cell.2016.04.041 27153494 PMC4871617

[94] Chrousos GP. The hypothalamic-pituitary-adrenal axis and immune-mediated inflammation. N Engl J Med. (1995) 332:1351–62. 10.1056/NEJM199505183322008 7715646

[95] Webster JI Tonelli L Sternberg EM. Neuroendocrine regulation of immunity. Annu Rev Immunol. (2002) 20:125–63. 10.1146/annurev.immunol.20.082401.104914 11861600

[96] Rivest S. Interactions between the immune and neuroendocrine systems. Prog Brain Res. (2010) 181:43–53. 10.1016/S0079-6123(08)81004-7 20478432

[97] Quatrini L Wieduwild E Escaliere B Filtjens J Chasson L Laprie C. Endogenous glucocorticoids control host resistance to viral infection through the tissue-specific regulation of PD-1 expression on NK cells. Nat Immunol. (2018) 19:954–62. 10.1038/s41590-018-0185-0 30127438 PMC6138242

[98] Crowley T Cryan JF Downer EJ O'Leary OF. Inhibiting neuroinflammation: The role and therapeutic potential of GABA in neuro-immune interactions. Brain Behav Immun. (2016) 54:260–77. 10.1016/j.bbi.2016.02.001 26851553

[99] Wu C Qin X Du H Li N Ren W Peng Y. The immunological function of GABAergic system. Front Biosci. (2017) 22:1162–72. 10.2741/4539 28199198

[100] Barragan A Weidner JM Jin Z Korpi ER Birnir B. GABAergic signalling in the immune system. Acta Physiol. (2015) 213:819–27. 10.1111/apha.12467 25677654

[101] Dunne JW Davidson L Vandongen R Beilin LJ Rogers P. The effect of ascorbic acid on plasma sulfate conjugated catecholamines after eating bananas. Life Sci. (1983) 33:1511–7. 10.1016/0024-3205(83)90855-X 6621254

[102] Barron AB Sovik E Cornish JL. The roles of dopamine and related compounds in reward-seeking behavior across animal phyla. Front Behav Neurosci. (2010) 4:163. 10.3389/fnbeh.2010.00163 21048897 PMC2967375

[103] Cottrell GA. Occurrence of dopamine and noradrenaline in the nervous tissue of some invertebrate species. Br J Pharmacol Chemother. (1967) 29:63–9. 10.1111/j.1476-5381.1967.tb01939.x 19108240 PMC1557178

[104] Levite M. Neurotransmitters activate T-cells and elicit crucial functions via neurotransmitter receptors. Curr Opin Pharmacol. (2008) 8:460–71. 10.1016/j.coph.2008.05.001 18579442

[105] Watanabe Y Nakayama T Nagakubo D Hieshima K Jin Z Katou F. Dopamine selectively induces migration and homing of naive CD8+ T cells via dopamine receptor D3. J Immunol. (2006) 176:848–56. 10.4049/jimmunol.176.2.848 16393968

[106] Bengel FM. Imaging targets of the sympathetic nervous system of the heart: translational considerations. J Nucl Med. (2011) 52:1167–70. 10.2967/jnumed.110.084228 21764793

[107] Puglisi-Allegra S Ventura R. Prefrontal/accumbal catecholamine system processes high motivational salience. Front Behav Neurosci. (2012) 6:31. 10.3389/fnbeh.2012.00031 22754514 PMC3384081

[108] Eisenhofer G Kopin IJ Goldstein DS. Catecholamine metabolism: a contemporary view with implications for physiology and medicine. Pharmacol Rev. (2004) 56:331–49. 10.1124/pr.56.3.1 15317907

[109] Sarkar C Basu B Chakroborty D Dasgupta PS Basu S. The immunoregulatory role of dopamine: an update. Brain Behav Immun. (2010) 24:525–8. 10.1016/j.bbi.2009.10.015 19896530 PMC2856781

[110] Cosentino M Fietta AM Ferrari M Rasini E Bombelli R Carcano E. Human CD4+CD25+ regulatory T cells selectively express tyrosine hydroxylase and contain endogenous catecholamines subserving an autocrine/paracrine inhibitory functional loop. Blood. (2007) 109:632–42. 10.1182/blood-2006-01-028423 16985181

[111] Cosentino M Bombelli R Ferrari M Marino F Rasini E Maestroni GJ. HPLC-ED measurement of endogenous catecholamines in human immune cells and hematopoietic cell lines. Life Sci. (2000) 68:283–95. 10.1016/S0024-3205(00)00937-1 11191644

[112] Nakano K Higashi T Takagi R Hashimoto K Tanaka Y Matsushita S. Dopamine released by dendritic cells polarizes Th2 differentiation. Int Immunol. (2009) 21:645–54. 10.1093/intimm/dxp033 19332443

[113] Borroto-Escuela DO Rodriguez D Romero-Fernandez W Kapla J Jaiteh M Ranganathan A. Mapping the interface of a GPCR dimer: a structural model of the A2A adenosine and D2 dopamine receptor heteromer. Front Pharmacol. (2018) 9:829. 10.3389/fphar.2018.00829 30214407 PMC6125358

[114] Borroto-Escuela DO Brito I Romero-Fernandez W Di Palma M Oflijan J Skieterska K. The G protein-coupled receptor heterodimer network (GPCR-HetNet) and its hub components. Int J Mol Sci. (2014) 15:8570–90. 10.3390/ijms15058570 24830558 PMC4057749

[115] Neve KA Seamans JK Trantham-Davidson H. Dopamine receptor signaling. J Recept Signal Transduct Res. (2004) 24:165–205. 10.1081/RRS-200029981 15521361

[116] Takahashi N Nagai Y Ueno S Saeki Y Yanagihara T. Human peripheral blood lymphocytes express D5 dopamine receptor gene and transcribe the two pseudogenes. FEBS Lett. (1992) 314:23–5. 10.1016/0014-5793(92)81452-R 1451800

[117] McKenna F McLaughlin PJ Lewis BJ Sibbring GC Cummerson JA Bowen-Jones D. Dopamine receptor expression on human T- and B-lymphocytes, monocytes, neutrophils, eosinophils and NK cells: a flow cytometric study. J Neuroimmunol. (2002) 132:34–40. 10.1016/S0165-5728(02)00280-1 12417431

[118] Nakano K Higashi T Hashimoto K Takagi R Tanaka Y Matsushita S. Antagonizing dopamine D1-like receptor inhibits Th17 cell differentiation: preventive and therapeutic effects on experimental autoimmune encephalomyelitis. Biochem Biophys Res Commun. (2008) 373:286–91. 10.1016/j.bbrc.2008.06.012 18558081

[119] Strell C Sievers A Bastian P Lang K Niggemann B Zanker KS. Divergent effects of norepinephrine, dopamine and substance P on the activation, differentiation and effector functions of human cytotoxic T lymphocytes. BMC Immunol. (2009) 10:62. 10.1186/1471-2172-10-62 19968887 PMC2794263

[120] Levite M Chowers Y Ganor Y Besser M Hershkovits R Cahalon L. Dopamine interacts directly with its D3 and D2 receptors on normal human T cells, and activates beta1 integrin function. Eur J Immunol. (2001) 31:3504–12. 10.1002/1521-4141(200112)31:12<3504::AID-IMMU3504>3.0.CO;2-F 11745370

[121] Kipnis J Cardon M Avidan H Lewitus GM Mordechay S Rolls A. Dopamine, through the extracellular signal-regulated kinase pathway, downregulates CD4+CD25+ regulatory T-cell activity: implications for neurodegeneration. J Neurosci. (2004) 24:6133–43. 10.1523/JNEUROSCI.0600-04.2004 15240805 PMC6729670

[122] Papa I Saliba D Ponzoni M Bustamante S Canete PF Gonzalez-Figueroa P. TFH-derived dopamine accelerates productive synapses in germinal centres. Nature. (2017) 547:318–23. 10.1038/nature23013 28700579 PMC5540173

[123] Vidal PM Pacheco R. Targeting the dopaminergic system in autoimmunity. J Neuroimmune Pharmacol. (2020) 15:57–73. 10.1007/s11481-019-09834-5 30661214

[124] Wang W Cohen JA Wallrapp A Trieu KG Barrios J Shao F. Age-related dopaminergic innervation augments T helper 2-type allergic inflammation in the postnatal lung. Immunity. (2019) 17:1102–18.e7. 10.2139/ssrn.3389887 31757673 PMC6937208

[125] Besser MJ Ganor Y Levite M. Dopamine by itself activates either D2, D3, or D1/D5 dopaminergic receptors in normal human T-cells and triggers the selective secretion of either IL-10, TNFalpha or both. J Neuroimmunol. (2005) 169:161–71. 10.1016/j.jneuroim.2005.07.013 16150496

[126] Bergquist J Tarkowski A Ekman R Ewing A. Discovery of endogenous catecholamines in lymphocytes and evidence for catecholamine regulation of lymphocyte function via an autocrine loop. Proc Natl Acad Sci USA. (1994) 91:12912–6. 10.1073/pnas.91.26.12912 7809145 PMC45550

[127] Ghosh MC Mondal AC Basu S Banerjee S Majumder J Bhattacharya D. Dopamine inhibits cytokine release and expression of tyrosine kinases, Lck and Fyn in activated T cells. Int Immunopharmacol. (2003) 3:1019–26. 10.1016/S1567-5769(03)00100-0 12810359

[128] Sarkar C Das S Chakroborty D Chowdhury UR Basu B Dasgupta PS. Cutting Edge: stimulation of dopamine D4 receptors induce T cell quiescence by up-regulating Kruppel-like factor-2 expression through inhibition of ERK1/ERK2 phosphorylation. J Immunol. (2006) 177:7525–9. 10.4049/jimmunol.177.11.7525 17114421

[129] Saha B Mondal AC Majumder J Basu S Dasgupta PS. Physiological concentrations of dopamine inhibit the proliferation and cytotoxicity of human CD4+ and CD8+ T cells *in vitro*: a receptor-mediated mechanism. Neuroimmunomodulation. (2001) 9:23–33. 10.1159/000049004 11435749

[130] Mikulak J Bozzo L Roberto A Pontarini E Tentorio P Hudspeth K. Dopamine inhibits the effector functions of activated NK cells via the upregulation of the D5 receptor. J Immunol. (2014) 193:2792–800. 10.4049/jimmunol.1401114 25127864

[131] Cosentino M Rasini E Colombo C Marino F Blandini F Ferrari M. Dopaminergic modulation of oxidative stress and apoptosis in human peripheral blood lymphocytes: evidence for a D1-like receptor-dependent protective effect. Free Radic Biol Med. (2004) 36:1233–40. 10.1016/j.freeradbiomed.2004.02.065 15110388

[132] Pacheco R Prado CE Barrientos MJ Bernales S. Role of dopamine in the physiology of T-cells and dendritic cells. J Neuroimmunol. (2009) 216:8–19. 10.1016/j.jneuroim.2009.07.018 19732962

[133] Kawano M Takagi R Kaneko A Matsushita S. Berberine is a dopamine D1- and D2-like receptor antagonist and ameliorates experimentally induced colitis by suppressing innate and adaptive immune responses. J Neuroimmunol. (2015) 289:43–55. 10.1016/j.jneuroim.2015.10.001 26616870

[134] Torres-Rosas R Yehia G Pena G Mishra P del Rocio Thompson-Bonilla M Moreno-Eutimio MA. Dopamine mediates vagal modulation of the immune system by electroacupuncture. Nat Med. (2014) 20:291–5. 10.1038/nm.3479 24562381 PMC3949155

[135] Yan F Wang L Shi Y Cao H Liu L Washington MK. Berberine promotes recovery of colitis and inhibits inflammatory responses in colonic macrophages and epithelial cells in DSS-treated mice. Am J Physiol Gastrointest Liver Physiol. (2012) 302:G504–14. 10.1152/ajpgi.00312.2011 22173918 PMC3311435

[136] Giorelli M Livrea P Trojano M. Dopamine fails to regulate activation of peripheral blood lymphocytes from multiple sclerosis patients: effects of IFN-beta. J Interferon Cytokine Res. (2005) 25:395–406. 10.1089/jir.2005.25.395 16022584

[137] Nakano K Yamaoka K Hanami K Saito K Sasaguri Y Yanagihara N. Dopamine induces IL-6-dependent IL-17 production via D1-like receptor on CD4 naive T cells and D1-like receptor antagonist SCH-23390 inhibits cartilage destruction in a human rheumatoid arthritis/SCID mouse chimera model. J Immunol. (2011) 186:3745–52. 10.4049/jimmunol.1002475 21307293

[138] Muller N. Inflammation in Schizophrenia: pathogenetic aspects and therapeutic considerations. Schizophr Bull. (2018) 44:973–82. 10.1093/schbul/sby024 29648618 PMC6101562

[139] Cao W Zheng H. Correction to: peripheral immune system in aging and Alzheimer's disease. Mol Neurodegener. (2018) 13:58. 10.1186/s13024-018-0290-4 30355319 PMC6201538

[140] Cao W Zheng H Peripheral immune system in aging and Alzheimer's disease. Mol Neurodegener. (2018) 13:51 10.1186/s13024-018-0284-2 30285785 PMC6169078

[141] Phani S Loike JD Przedborski S. Neurodegeneration and inflammation in Parkinson's disease. Parkinsonism Relat Disord. (2012) 18(Suppl.1):S207–9. 10.1016/S1353-8020(11)70064-5 22166436

[142] Boneberg EM von Seydlitz E Propster K Watzl H Rockstroh B Illges H. D3 dopamine receptor mRNA is elevated in T cells of schizophrenic patients whereas D4 dopamine receptor mRNA is reduced in CD4+ -T cells. J Neuroimmunol. (2006) 173:180–7. 10.1016/j.jneuroim.2005.11.018 16376996

[143] Barbanti P Fabbrini G Ricci A Bruno G Cerbo R Bronzetti E. Luigi Lenzi: reduced density of dopamine D2-like receptors on peripheral blood lymphocytes in Alzheimer's disease. Mech Ageing Dev. (2000) 120:65–75. 10.1016/S0047-6374(00)00183-4 11087905

[144] Brochard V Combadiere B Prigent A Laouar Y Perrin A Beray-Berthat V. Infiltration of CD4+ lymphocytes into the brain contributes to neurodegeneration in a mouse model of Parkinson disease. J Clin Invest. (2009) 119:182–92. 10.1172/JCI36470 19104149 PMC2613467

[145] Gonzalez H Contreras F Prado C Elgueta D Franz D Bernales S. Dopamine receptor D3 expressed on CD4+ T cells favors neurodegeneration of dopaminergic neurons during Parkinson's disease. J Immunol. (2013) 190:5048–56. 10.4049/jimmunol.1203121 23589621

[146] Barbanti P Fabbrini G Ricci A Cerbo R Bronzetti E Caronti B. Increased expression of dopamine receptors on lymphocytes in Parkinson's disease. Mov Disord. (1999) 14:764–71. 10.1002/1531-8257(199909)14:5<764::AID-MDS1008>3.0.CO;2-W 10495037

[147] Orr CF Rowe DB Mizuno Y Mori H Halliday GM. A possible role for humoral immunity in the pathogenesis of Parkinson's disease. Brain. (2005) 128:2665–74. 10.1093/brain/awh625 16219675

[148] Fiszer U. Does Parkinson's disease have an immunological basis? The evidence and its therapeutic implications. BioDrugs. (2001) 15:351–5. 10.2165/00063030-200115060-00001 11520246

[149] Ciesielska A Joniec I Przybylkowski A Gromadzka G Kurkowska-Jastrzebska I Czlonkowska A. Dynamics of expression of the mRNA for cytokines and inducible nitric synthase in a murine model of the Parkinson's disease. Acta Neurobiol Exp. (2003) 63:117–26. 12926538 10.55782/ane-2003-1461

[150] Kurkowska-Jastrzebska I Wronska A Kohutnicka M Czlonkowski A Czlonkowska A. MHC class II positive microglia and lymphocytic infiltration are present in the substantia nigra and striatum in mouse model of Parkinson's disease. Acta Neurobiol Exp. (1999) 59:1–8. 10230070 10.55782/ane-1999-1289

[151] Huser A Rohwedder A Apostolopoulou AA Widmann A Pfitzenmaier JE Maiolo EM. The serotonergic central nervous system of the *Drosophila larva*: anatomy and behavioral function. PLoS ONE. (2012) 7:e47518. 10.1371/journal.pone.0047518 23082175 PMC3474743

[152] Feldman JM Lee EM Castleberry CA. Catecholamine and serotonin content of foods: effect on urinary excretion of homovanillic and 5-hydroxyindoleacetic acid. J Am Diet Assoc. (1987) 87:1031–5. 2440934

[153] Cools R Roberts AC Robbins TW. Serotoninergic regulation of emotional and behavioural control processes. Trends Cogn Sci. (2008) 12:31–40. 10.1016/j.tics.2007.10.011 18069045

[154] Andrews PW Bharwani A Lee KR Fox M Thomson JA Jr Is serotonin an upper or a downer? The evolution of the serotonergic system and its role in depression and the antidepressant response. Neurosci Biobehav Rev. (2015) 51:164–88. 10.1016/j.neubiorev.2015.01.018 25625874

[155] Carhart-Harris RL Nutt DJ. Serotonin and brain function: a tale of two receptors. J Psychopharmacol. (2017) 31:1091–120. 10.1177/0269881117725915 28858536 PMC5606297

[156] Gershon MD Drakontides AB Ross LL. Serotonin: synthesis and release from the myenteric plexus of the mouse intestine. Science. (1965) 149:197–9. 10.1126/science.149.3680.197 14305120

[157] Spohn SN Mawe GM. Non-conventional features of peripheral serotonin signalling - the gut and beyond. Nat Rev Gastroenterol Hepatol. (2017) 14:412–20. 10.1038/nrgastro.2017.51 28487547 PMC5672796

[158] Berger M Gray JA Roth BL. The expanded biology of serotonin. Annu Rev Med. (2009) 60:355–66. 10.1146/annurev.med.60.042307.110802 19630576 PMC5864293

[159] McGowan K Kane A Asarkof N Wicks J Guerina V Kellum J. Entamoeba histolytica causes intestinal secretion: role of serotonin. Science. (1983) 221:762–4. 10.1126/science.6308760 6308760

[160] Bouagnon AD Lin L Srivastava S Liu CC Panda O Schroeder FC. Intestinal peroxisomal fatty acid beta-oxidation regulates neural serotonin signaling through a feedback mechanism. PLoS Biol. (2019) 17:e3000242. 10.1371/journal.pbio.3000242 31805041 PMC6917301

[161] Srinivasan S Srinivasan U Balwani H. Effect of thyroxine, reerpine and serotonin on allyl alcohol induced hepatotoxicity in rats. Acta Pharmacol Toxicol. (1970) 28:338–45. 10.1111/j.1600-0773.1970.tb00560.x 5536734

[162] Huber R Orzeszyna M Pokorny N Kravitz EA. Biogenic amines and aggression: experimental approaches in crustaceans. Brain Behav Evol. (1997) 50(Suppl.1):60–8. 10.1159/000113355 9217993

[163] Kravitz EA. Hormonal control of behavior: amines and the biasing of behavioral output in lobsters. Science. (1988) 241:1775–81. 10.1126/science.2902685 2902685

[164] Ahern GP. 5-HT and the immune system. Curr Opin Pharmacol. (2011) 11:29–33. 10.1016/j.coph.2011.02.004 21393060 PMC3144148

[165] Baganz NL Blakely RD. A dialogue between the immune system and brain, spoken in the language of serotonin. ACS Chem Neurosci. (2013) 4:48–63. 10.1021/cn300186b 23336044 PMC3547518

[166] O'Connell PJ Wang X Leon-Ponte M Griffiths C Pingle SC Ahern GP. A novel form of immune signaling revealed by transmission of the inflammatory mediator serotonin between dendritic cells and T cells. Blood. (2006) 107:1010–7. 10.1182/blood-2005-07-2903 16223770 PMC1895901

[167] Nakamura K Sato T Ohashi A Tsurui H Hasegawa H. Role of a serotonin precursor in development of gut microvilli. Am J Pathol. (2008) 172:333–44. 10.2353/ajpath.2008.070358 18202184 PMC2312355

[168] Millan MJ Marin P Bockaert JC. Mannoury la cour: signaling at G-protein-coupled serotonin receptors: recent advances and future research directions. Trends Pharmacol Sci. (2008) 29:454–64. 10.1016/j.tips.2008.06.007 18676031

[169] Idzko M Panther E Stratz C Muller T Bayer H Zissel G. The serotoninergic receptors of human dendritic cells: identification and coupling to cytokine release. J Immunol. (2004) 172:6011–9. 10.4049/jimmunol.172.10.6011 15128784

[170] Nichols DE Nichols CD. Serotonin receptors. Chem Rev. (2008) 108:1614–41. 10.1021/cr078224o 18476671

[171] Herr N Bode C Duerschmied D. The effects of serotonin in immune cells. Front Cardiovasc Med. (2017) 4:48. 10.3389/fcvm.2017.00048 28775986 PMC5517399

[172] Kushnir-Sukhov NM Gilfillan AM Coleman JW Brown JM Bruening S Toth M. Metcalfe: 5-hydroxytryptamine induces mast cell adhesion and migration. J Immunol. (2006) 177:6422–32. 10.4049/jimmunol.177.9.6422 17056574

[173] Muller T Durk T Blumenthal B Grimm M Cicko S Panther E. Idzko: 5-hydroxytryptamine modulates migration, cytokine and chemokine release and T-cell priming capacity of dendritic cells *in vitro* and *in vivo*. PLoS ONE. (2009) 4:e6453. 10.1371/journal.pone.0006453 19649285 PMC2714071

[174] Durk T Panther E Muller T Sorichter S Ferrari D Pizzirani C. 5-Hydroxytryptamine modulates cytokine and chemokine production in LPS-primed human monocytes via stimulation of different 5-HTR subtypes. Int Immunol. (2005) 17:599–606. 10.1093/intimm/dxh242 15802305

[175] Boehme SA Lio FM Sikora L Pandit TS Lavrador K Rao SP. Cutting edge: serotonin is a chemotactic factor for eosinophils and functions additively with eotaxin. J Immunol. (2004) 173:3599–603. 10.4049/jimmunol.173.6.3599 15356103

[176] Mikulski Z Zaslona Z Cakarova L Hartmann P Wilhelm J Tecott LH. Serotonin activates murine alveolar macrophages through 5-HT2C receptors. Am J Physiol Lung Cell Mol Physiol. (2010) 299:L272–80. 10.1152/ajplung.00032.2010 20495077

[177] Yin J Albert RH Tretiakova AP Jameson BA. 5-HT(1B) receptors play a prominent role in the proliferation of T-lymphocytes. J Neuroimmunol. (2006) 181:68–81. 10.1016/j.jneuroim.2006.08.004 17011639

[178] Inoue M Okazaki T Kitazono T Mizushima M Omata M Ozaki S. Regulation of antigen-specific CTL and Th1 cell activation through 5-Hydroxytryptamine 2A receptor. Int Immunopharmacol. (2011) 11:67–73. 10.1016/j.intimp.2010.10.007 20971187

[179] Rinaldi A Chiaravalli AM Mian M Zucca E Tibiletti MG Capella C. Serotonin receptor 3A expression in normal and neoplastic B cells. Pathobiology. (2010) 77:129–35. 10.1159/000292646 20516728

[180] Mossner R Lesch KP. Role of serotonin in the immune system and in neuroimmune interactions. Brain Behav Immun. (1998) 12:249–71. 10.1006/brbi.1998.0532 10080856

[181] Choquet D Korn H. Dual effects of serotonin on a voltage-gated conductance in lymphocytes. Proc Natl Acad Sci USA. (1988) 85:4557–61. 10.1073/pnas.85.12.4557 3260036 PMC280470

[182] Xiao J Shao L Shen J Jiang W Feng Y Zheng P. Effects of ketanserin on experimental colitis in mice and macrophage function. Int J Mol Med. (2016) 37:659–68. 10.3892/ijmm.2016.2486 26865503 PMC4771115

[183] Nau F Jr Miller J Saravia J Ahlert T Yu B Happel KI. Serotonin 5-HT(2) receptor activation prevents allergic asthma in a mouse model. Am J Physiol Lung Cell Mol Physiol. (2015) 308:L191–8. 10.1152/ajplung.00138.2013 25416380 PMC4338939

[184] Yu B Becnel J Zerfaoui M Rohatgi R Boulares AH Nichols CD. Serotonin 5-hydroxytryptamine(2A) receptor activation suppresses tumor necrosis factor-alpha-induced inflammation with extraordinary potency. J Pharmacol Exp Ther. (2008) 327:316–23. 10.1124/jpet.108.143461 18708586

[185] Chabbi-Achengli Y Coman T Collet C Callebert J Corcelli M Lin H. Serotonin is involved in autoimmune arthritis through Th17 immunity and bone resorption. Am J Pathol. (2016) 186:927–37. 10.1016/j.ajpath.2015.11.018 26968113

[186] Sacramento PM Monteiro C Dias ASO Kasahara TM Ferreira TB Hygino J. Serotonin decreases the production of Th1/Th17 cytokines and elevates the frequency of regulatory CD4(+) T-cell subsets in multiple sclerosis patients. Eur J Immunol. (2018) 48:1376–88. 10.1002/eji.201847525 29719048

[187] Dubayle D Serviere J Menetrey D. Evidence for serotonin influencing the thalamic infiltration of mast cells in rat. J Neuroimmunol. (2005) 159:20–30. 10.1016/j.jneuroim.2004.08.020 15652399

[188] Walther A Peter C Yilmaz N Schmidt W Martin E Schmidt H. Influence of serotonin-receptor antagonism on mast cell activation during endotoxemia. Pathophysiology. (2002) 8:161–5. 10.1016/S0928-4680(02)00006-8 12039647

[189] Mahe C Loetscher E Dev KK Bobirnac I Otten U Schoeffter P. Serotonin 5-HT7 receptors coupled to induction of interleukin-6 in human microglial MC-3 cells. Neuropharmacology. (2005) 49:40–7. 10.1016/j.neuropharm.2005.01.025 15992579

[190] Freire-Garabal M Nunez MJ Balboa J Lopez-Delgado P Gallego R Garcia-Caballero T. Serotonin upregulates the activity of phagocytosis through 5-HT1A receptors. Br J Pharmacol. (2003) 139:457–63. 10.1038/sj.bjp.0705188 12770951 PMC1573834

[191] Maneglier B Guillemin GJ Clayette P Rogez-Kreuz C Brew BJ Dormont D. Serotonin decreases HIV-1 replication in primary cultures of human macrophages through 5-HT(1A) receptors. Br J Pharmacol. (2008) 154:174–82. 10.1038/bjp.2008.80 18332855 PMC2438982

[192] Sadiq A Shah A Jeschke MG Belo C Qasim Hayat M Murad S. The role of serotonin during skin healing in post-thermal injury. Int J Mol Sci. (2018) 19:1034. 10.3390/ijms19041034 29596386 PMC5979562

[193] Kenis G Maes M Effects of antidepressants on the production of cytokines. Int J Neuropsychopharmacol. (2002) 5:401–12. 10.1017/S1461145702003164 12466038

[194] Wang L Wang R Liu L Qiao D Baldwin DS Hou R. Effects of SSRIs on peripheral inflammatory markers in patients with major depressive disorder: a systematic review and meta-analysis. Brain Behav Immun. (2019) 79:24–38. 10.1016/j.bbi.2019.02.021 30797959

[195] Nazimek K Strobel S Bryniarski P Kozlowski M Filipczak-Bryniarska I Bryniarski K. The role of macrophages in anti-inflammatory activity of antidepressant drugs. Immunobiology. (2017) 222:823–30. 10.1016/j.imbio.2016.07.001 27453459

[196] Gobin V Van Steendam K Denys D Deforce D. Selective serotonin reuptake inhibitors as a novel class of immunosuppressants. Int Immunopharmacol. (2014) 20:148–56. 10.1016/j.intimp.2014.02.030 24613205

[197] Sluzewska A Rybakowski JK Laciak M Mackiewicz A Sobieska M Wiktorowicz K. Interleukin-6 serum levels in depressed patients before and after treatment with fluoxetine. Ann N Y Acad Sci. (1995) 762:474–6. 10.1111/j.1749-6632.1995.tb32372.x 7668562

[198] Basterzi AD Aydemir C Kisa C Aksaray S Tuzer V Yazici K. IL-6 levels decrease with SSRI treatment in patients with major depression. Hum Psychopharmacol. (2005) 20:473–6. 10.1002/hup.717 16158446

[199] Lanquillon S Krieg JC Bening-Abu-Shach U Vedder H. Cytokine production and treatment response in major depressive disorder. Neuropsychopharmacology. (2000) 22:370–9. 10.1016/S0893-133X(99)00134-7 10700656

[200] Roumestan C Michel A Bichon F Portet K Detoc M Henriquet C. Anti-inflammatory properties of desipramine and fluoxetine. Respir Res. (2007) 8:35. 10.1186/1465-9921-8-35 17477857 PMC1876225

[201] Nazimek K Kozlowski M Bryniarski P Strobel S Bryk A Myszka M. Repeatedly administered antidepressant drugs modulate humoral and cellular immune response in mice through action on macrophages. Exp Biol Med. (2016) 241:1540–50. 10.1177/1535370216643769 27053354 PMC4994903

[202] Choquet D Korn H. Modulation of voltage-dependent potassium channels in B lymphocytes. Biochem Pharmacol. (1988) 37:3797–802. 10.1016/0006-2952(88)90058-5 3056414

[203] Maes M. Major depression and activation of the inflammatory response system. Adv Exp Med Biol. (1999) 461:25–46. 10.1007/978-0-585-37970-8_2 10442165

[204] Muller N Schwarz MJ. The immune-mediated alteration of serotonin and glutamate: towards an integrated view of depression. Mol Psychiatry. (2007) 12:988–1000. 10.1038/sj.mp.4002006 17457312

[205] Dowlati Y Herrmann N Swardfager W Liu H Sham L Reim EK. A meta-analysis of cytokines in major depression. Biol Psychiatry. (2010) 67:446–57. 10.1016/j.biopsych.2009.09.033 20015486

[206] Hesse S Moeller F Petroff D Lobsien D Luthardt J Regenthal R. Altered serotonin transporter availability in patients with multiple sclerosis. Eur J Nucl Med Mol Imaging. (2014) 41:827–35. 10.1007/s00259-013-2636-z 24562640

[207] Spiller R. Serotonin and GI clinical disorders. Neuropharmacology. (2008) 55:1072–80. 10.1016/j.neuropharm.2008.07.016 18687345

[208] Mawe GM Hoffman JM. Serotonin signalling in the gut–functions, dysfunctions and therapeutic targets. Nat Rev Gastroenterol Hepatol. (2013) 10:473–86. 10.1038/nrgastro.2013.105 23797870 PMC4048923

[209] Spiller RC. Inflammation as a basis for functional GI disorders. Best Pract Res Clin Gastroenterol. (2004) 18:641–61. 10.1016/j.bpg.2004.04.002 15324705

[210] Jin DC Cao HL Xu MQ Wang SN Wang YM Yan F. Regulation of the serotonin transporter in the pathogenesis of irritable bowel syndrome. World J Gastroenterol. (2016) 22:8137–48. 10.3748/wjg.v22.i36.8137 27688655 PMC5037082

[211] Sikander A Rana SV Prasad KK. Role of serotonin in gastrointestinal motility and irritable bowel syndrome. Clin Chim Acta. (2009) 403:47–55. 10.1016/j.cca.2009.01.028 19361459

[212] Shajib MS Wang H Kim JJ Sunjic I Ghia JE Denou E. Interleukin 13 and serotonin: linking the immune and endocrine systems in murine models of intestinal inflammation. PLoS ONE. (2013) 8:e72774. 10.1371/journal.pone.0072774 24015275 PMC3755966

[213] Fayyaz M Lackner JM. Serotonin receptor modulators in the treatment of irritable bowel syndrome. Ther Clin Risk Manag. (2008) 4:41–8. 10.2147/TCRM.S140 18728719 PMC2503665

[214] Wan M Ding L Wang D Han J Gao P. Serotonin: a potent immune cell modulator in autoimmune diseases. Front Immunol. (2020) 11:186. 10.3389/fimmu.2020.00186 32117308 PMC7026253

[215] Masri MFB Mantri CK Rathore APS St. John AL. Peripheral serotonin causes dengue-induced thrombocytopenia through 5HT. Blood. (2019) 133:2325–37. 10.1182/blood-2018-08-869156 30755421

[216] Uyttenhove C Pilotte L Theate I Stroobant V Colau D Parmentier N. Evidence for a tumoral immune resistance mechanism based on tryptophan degradation by indoleamine 2,3-dioxygenase. Nat Med. (2003) 9:1269–74. 10.1038/nm934 14502282

[217] de Aquino MTP Malhotra A Mishra MK Shanker A. Challenges and future perspectives of T cell immunotherapy in cancer. Immunol Lett. (2015) 166:117–33. 10.1016/j.imlet.2015.05.018 26096822 PMC4499494

[218] Cunin P Caillon A Corvaisier M Garo E Scotet M Blanchard S. The tachykinins substance P and hemokinin-1 favor the generation of human memory Th17 cells by inducing IL-1beta, IL-23, and TNF-like 1A expression by monocytes. J Immunol. (2011) 186:4175–82. 10.4049/jimmunol.1002535 21368235

[219] O'Connor TM O'Connell J O'Brien DI Goode T Bredin CP Shanahan F. The role of substance P in inflammatory disease. J Cell Physiol. (2004) 201:167–80. 10.1002/jcp.20061 15334652

[220] Suvas S. Role of substance P neuropeptide in inflammation, wound healing, tissue homeostasis. J Immunol. (2017) 199:1543–52. 10.4049/jimmunol.1601751 28827386 PMC5657331

[221] Gerard NP Garraway LA Eddy RL Jr Shows TB Iijima H Paquet JL. Human substance P receptor (NK-1): organization of the gene, chromosome localization, and functional expression of cDNA clones. Biochemistry. (1991) 30:10640–6. 10.1021/bi00108a006 1657150

[222] Gobbi G Cassano T Radja F Morgese MG Cuomo V Santarelli L. Neurokinin 1 receptor antagonism requires norepinephrine to increase serotonin function. Eur Neuropsychopharmacol. (2007) 17:328–38. 10.1016/j.euroneuro.2006.07.004 16950604

[223] Kovacs KA Steinmann M Magistretti PJ Halfon O Cardinaux JR. C/EBPbeta couples dopamine signalling to substance P precursor gene expression in striatal neurones. J Neurochem. (2006) 98:1390–9. 10.1111/j.1471-4159.2006.03957.x 16771829

[224] Rameshwar P. Substance P: a regulatory neuropeptide for hematopoiesis and immune functions. Clin Immunol Immunopathol. (1997) 85:129–33. 10.1006/clin.1997.4446 9344694

[225] Pinto FM Almeida TA Hernandez M Devillier P Advenier C Candenas ML. mRNA expression of tachykinins and tachykinin receptors in different human tissues. Eur J Pharmacol. (2004) 494:233–9. 10.1016/j.ejphar.2004.05.016 15212980

[226] Nessler S Stadelmann C Bittner A Schlegel K Gronen F Brueck W. Suppression of autoimmune encephalomyelitis by a neurokinin-1 receptor antagonist–a putative role for substance P in CNS inflammation. J Neuroimmunol. (2006) 179:1–8. 10.1016/j.jneuroim.2006.06.026 16904192

[227] Ho WZ Douglas SD. Substance P and neurokinin-1 receptor modulation of HIV. J Neuroimmunol. (2004) 157:48–55. 10.1016/j.jneuroim.2004.08.022 15579279

[228] Lambert N Lescoulie PL Yassine-Diab B Enault G Mazieres B De Preval C. Substance P enhances cytokine-induced vascular cell adhesion molecule-1 (VCAM-1) expression on cultured rheumatoid fibroblast-like synoviocytes. Clin Exp Immunol. (1998) 113:269–75. 10.1046/j.1365-2249.1998.00621.x 9717978 PMC1905034

[229] Azzolina A Bongiovanni A Lampiasi N. Substance P induces TNF-alpha and IL-6 production through NF kappa B in peritoneal mast cells. Biochim Biophys Acta. (2003) 1643:75–83. 10.1016/j.bbamcr.2003.09.003 14654230

[230] Azzolina A Guarneri P Lampiasi N. Involvement of p38 and JNK MAPKs pathways in Substance P-induced production of TNF-alpha by peritoneal mast cells. Cytokine. (2002) 18:72–80. 10.1006/cyto.2002.0879 12096921

[231] Donkin JJ Turner RJ Hassan I Vink R. Substance P in traumatic brain injury. Prog Brain Res. (2007) 161:97–109. 10.1016/S0079-6123(06)61007-8 17618972

[232] Lambrecht BN Germonpre PR Everaert EG Carro-Muino I de Veerman M de Felipe C. Endogenously produced substance P contributes to lymphocyte proliferation induced by dendritic cells and direct TCR ligation. Eur J Immunol. (1999) 29:3815–25. 10.1002/(SICI)1521-4141(199912)29:12<3815::AID-IMMU3815>3.0.CO;2-# 10601989

[233] Monaco-Shawver L Schwartz L Tuluc F Guo CJ Lai JP Gunnam SM. Substance P inhibits natural killer cell cytotoxicity through the neurokinin-1 receptor. J Leukoc Biol. (2011) 89:113–25. 10.1189/jlb.0410200 20940324 PMC3004520

[234] Mashaghi A Marmalidou A Tehrani M Grace PM Pothoulakis C Dana R. Neuropeptide substance P and the immune response. Cell Mol Life Sci. (2016) 73:4249–64. 10.1007/s00018-016-2293-z 27314883 PMC5056132

[235] DeFea KA Vaughn ZD O'Bryan EM Nishijima D Dery O Bunnett NW. The proliferative and antiapoptotic effects of substance P are facilitated by formation of a beta -arrestin-dependent scaffolding complex. Proc Natl Acad Sci USA. (2000) 97:11086–91. 10.1073/pnas.190276697 10995467 PMC27152

[236] Douglas SD Leeman SE. Neurokinin-1 receptor: functional significance in the immune system in reference to selected infections and inflammation. Ann N Y Acad Sci. (2011) 1217:83–95. 10.1111/j.1749-6632.2010.05826.x 21091716 PMC3058850

[237] Nadel JA Borson DB. Modulation of neurogenic inflammation by neutral endopeptidase. Am Rev Respir Dis. (1991) 143:S33–6. 10.1164/ajrccm/143.3_Pt_2.S33 2003687

[238] Sun J Ramnath RD Bhatia M. Neuropeptide substance P upregulates chemokine and chemokine receptor expression in primary mouse neutrophils. Am J Physiol Cell Physiol. (2007) 293:C696–704. 10.1152/ajpcell.00060.2007 17494633

[239] Kuo HP Lin HC Hwang KH Wang CH Lu LC. Lipopolysaccharide enhances substance P-mediated neutrophil adherence to epithelial cells and cytokine release. Am J Respir Crit Care Med. (2000) 162:1891–7. 10.1164/ajrccm.162.5.9911065 11069831

[240] Tripp RA Barskey A Goss L Anderson LJ. Substance P receptor expression on lymphocytes is associated with the immune response to respiratory syncytial virus infection. J Neuroimmunol. (2002) 129:141–53. 10.1016/S0165-5728(02)00169-8 12161030

[241] Dusser DJ Djokic TD Borson DB Nadel JA. Cigarette smoke induces bronchoconstrictor hyperresponsiveness to substance P and inactivates airway neutral endopeptidase in the guinea pig. Possible role of free radicals. J Clin Invest. (1989) 84:900–6. 10.1172/JCI114251 2474576 PMC329734

[242] Ikeda Y Takei H Matsumoto C Mase A Yamamoto M Takeda S. Administration of substance P during a primary immune response amplifies the secondary immune response via a long-lasting effect on CD8+ T lymphocytes. Arch Dermatol Res. (2007) 299:345–51. 10.1007/s00403-007-0767-4 17643253

[243] Zhang Z Zheng W Xie H Chai R Wang J Zhang H. Up-regulated expression of substance P in CD8(+) T cells and NK1R on monocytes of atopic dermatitis. J Transl Med. (2017) 15:93. 10.1186/s12967-017-1196-6 28460633 PMC5412038

[244] Sandoval-Talamantes AK Gomez-Gonzalez BA Uriarte-Mayorga DF Martinez-Guzman MA Wheber-Hidalgo KA Alvarado-Navarro A. Neurotransmitters, neuropeptides and their receptors interact with immune response in healthy and psoriatic skin. Neuropeptides. (2020) 79:102004. 10.1016/j.npep.2019.102004 31902596

[245] Krause JE DiMaggio DA McCarson KE. Alterations in neurokinin 1 receptor gene expression in models of pain and inflammation. Can J Physiol Pharmacol. (1995) 73:854–9. 10.1139/y95-117 8846421

[246] Levine JD Moskowitz MA Basbaum AI. The contribution of neurogenic inflammation in experimental arthritis. J Immunol. (1985) 135:843s–7. 2409171

[247] Um J Jung N Kim D Choi S Lee SH Son Y. Substance P preserves pancreatic β-cells in type 1 and type 2 diabetic mice. Biochem Biophys Res Commun. (2018) 499:960–6. 10.1016/j.bbrc.2018.04.028 29626466

[248] Berger M Neth O Ilmer M Garnier A Salinas-Martin MV de Agustin Asencio JC. Hepatoblastoma cells express truncated neurokinin-1 receptor and can be growth inhibited by aprepitant *in vitro* and *in vivo*. J Hepatol. (2014) 60:985–94. 10.1016/j.jhep.2013.12.024 24412605

[249] Moroz LL Kocot KM Citarella MR Dosung S Norekian TP Povolotskaya IS. The ctenophore genome and the evolutionary origins of neural systems. Nature. (2014) 510:109–14. 10.1038/nature13400 24847885 PMC4337882

[250] Meldrum BS. Glutamate as a neurotransmitter in the brain: review of physiology and pathology. J Nutr. (2000) 130(4S Suppl.):1007S–15. 10.1093/jn/130.4.1007S 10736372

[251] McEntee WJ Crook TH. Glutamate: its role in learning, memory, and the aging brain. Psychopharmacology. (1993) 111:391–401. 10.1007/BF02253527 7870979

[252] Hayashi T. A physiological study of epileptic seizures following cortical stimulation in animals and its application to human clinics. Jpn J Physiol. (1952) 3:46–64. 10.2170/jjphysiol.3.46 13034377

[253] Ganor Y Levite M The neurotransmitter glutamate and human T cells: glutamate receptors and glutamate-induced direct and potent effects on normal human T cells, cancerous human leukemia and lymphoma T cells, and autoimmune human T cells. J Neural Transm. (2014) 121:983–1006. 10.1007/s00702-014-1167-5 24584970

[254] Smith QR. Transport of glutamate and other amino acids at the blood-brain barrier. J Nutr. (2000)130(4S Suppl.):1016S–22. 10.1093/jn/130.4.1016S 10736373

[255] Pin JP Duvoisin R. The metabotropic glutamate receptors: structure and functions. Neuropharmacology. (1995) 34:1–26. 10.1016/0028-3908(94)00129-G 7623957

[256] Fallarino F Volpi C Fazio F Notartomaso S Vacca C Busceti C. Di Marco: metabotropic glutamate receptor-4 modulates adaptive immunity and restrains neuroinflammation. Nat Med. (2010) 16:897–902. 10.1038/nm.2183 20657581

[257] Kritis AA Stamoula EG Paniskaki KA Vavilis TD. Researching glutamate - induced cytotoxicity in different cell lines: a comparative/collective analysis/study. Front Cell Neurosci. (2015) 9:91. 10.3389/fncel.2015.00091 25852482 PMC4362409

[258] Eisenkraft A Falk A Finkelstein A. The role of glutamate and the immune system in organophosphate-induced CNS damage. Neurotox Res. (2013) 24:265–79. 10.1007/s12640-013-9388-1 23532600

[259] Evonuk KS Baker BJ Doyle RE Moseley CE Sestero CM Johnston BP. Inhibition of system Xc(-) transporter attenuates autoimmune inflammatory demyelination. J Immunol. (2015) 195:450–63. 10.4049/jimmunol.1401108 26071560 PMC4490999

[260] Sexton RE Hachem AH Assi AA Bukhsh MA Gorski DH Speyer CL. Metabotropic glutamate receptor-1 regulates inflammation in triple negative breast cancer. Sci Rep. (2018) 8:16008. 10.1038/s41598-018-34502-8 30375476 PMC6207734

[261] Pacheco R Ciruela F Casado V Mallol J Gallart T Lluis C. Group I metabotropic glutamate receptors mediate a dual role of glutamate in T cell activation. J Biol Chem. (2004) 279:33352–8. 10.1074/jbc.M401761200 15184389

[262] Ganor Y Teichberg VI Levite M. TCR activation eliminates glutamate receptor GluR3 from the cell surface of normal human T cells, via an autocrine/paracrine granzyme B-mediated proteolytic cleavage. J Immunol. (2007) 178:683–92. 10.4049/jimmunol.178.2.683 17202328

[263] Orihara K Odemuyiwa SO Stefura WP Ilarraza R HayGlass KT Moqbel R. Neurotransmitter signalling via NMDA receptors leads to decreased T helper type 1-like and enhanced T helper type 2-like immune balance in humans. Immunology. (2018) 153:368–79. 10.1111/imm.12846 28940416 PMC5795186

[264] Lin CS Boltz RC Blake JT Nguyen M Talento A Fischer PA. Voltage-gated potassium channels regulate calcium-dependent pathways involved in human T lymphocyte activation. J Exp Med. (1993) 177:637–45. 10.1084/jem.177.3.637 7679705 PMC2190940

[265] Komada H Nakabayashi H Hara M Izutsu K. Early calcium signaling and calcium requirements for the IL-2 receptor expression and IL-2 production in stimulated lymphocytes. Cell Immunol. (1996) 173:215–20. 10.1006/cimm.1996.0270 8912879

[266] Lombardi G Dianzani C Miglio G Canonico PL Fantozzi R. Characterization of ionotropic glutamate receptors in human lymphocytes. Br J Pharmacol. (2001) 133:936–44. 10.1038/sj.bjp.0704134 11454668 PMC1572842

[267] Poulopoulou C Markakis I Davaki P Nikolaou C Poulopoulos A Raptis E. Modulation of voltage-gated potassium channels in human T lymphocytes by extracellular glutamate. Mol Pharmacol. (2005) 67:856–67. 10.1124/mol.67.3.856 15718225

[268] Chiocchetti A Miglio G Mesturini R Varsaldi F Mocellin M Orilieri E. Group I mGlu receptor stimulation inhibits activation-induced cell death of human T lymphocytes. Br J Pharmacol. (2006) 148:760–8. 10.1038/sj.bjp.0706746 16751798 PMC1617076

[269] Green DR Droin N Pinkoski M Activation-induced cell death in T cells. Immunol Rev. (2003) 193:70–81. 10.1034/j.1600-065X.2003.00051.x 12752672

[270] Pacheco R Gallart T Lluis C Franco R. Role of glutamate on T-cell mediated immunity. J Neuroimmunol. (2007) 185:9–19. 10.1016/j.jneuroim.2007.01.003 17303252

[271] Johnson MO Wolf MM Madden MZ Andrejeva G Sugiura A Contreras DC. Distinct regulation of Th17 and Th1 cell differentiation by glutaminase-dependent metabolism. Cell. (2018) 175:1780–95 e19. 10.1016/j.cell.2018.10.001 30392958 PMC6361668

[272] Levite M. Autoimmune epilepsy. Nat Immunol. (2002) 3:500. 10.1038/ni0602-500 12032558

[273] Pacheco R Oliva H Martinez-Navio JM Climent N Ciruela F Gatell JM. Glutamate released by dendritic cells as a novel modulator of T cell activation. J Immunol. (2006) 177:6695–704. 10.4049/jimmunol.177.10.6695 17082582

[274] Zerangue N Kavanaugh MP. Flux coupling in a neuronal glutamate transporter. Nature. (1996) 383:634–7. 10.1038/383634a0 8857541

[275] Miglio G Varsaldi F Lombardi G. Human T lymphocytes express N-methyl-D-aspartate receptors functionally active in controlling T cell activation. Biochem Biophys Res Commun. (2005) 338:1875–83. 10.1016/j.bbrc.2005.10.164 16289038

[276] Sinclair LV Rolf J Emslie E Shi YB Taylor PM Cantrell DA. Control of amino-acid transport by antigen receptors coordinates the metabolic reprogramming essential for T cell differentiation. Nat Immunol. (2013) 14:500–8. 10.1038/ni.2556 23525088 PMC3672957

[277] Nakaya M Xiao Y Zhou X Chang JH Chang M Cheng X. Inflammatory T cell responses rely on amino acid transporter ASCT2 facilitation of glutamine uptake and mTORC1 kinase activation. Immunity. (2014) 40:692–705. 10.1016/j.immuni.2014.04.007 24792914 PMC4074507

[278] Wang JB Erickson JW Fuji R Ramachandran S Gao P Dinavahi R. Targeting mitochondrial glutaminase activity inhibits oncogenic transformation. Cancer Cell. (2010) 18:207–19. 10.1016/j.ccr.2010.08.009 20832749 PMC3078749

[279] Boldyrev AA Carpenter DO Johnson P. Emerging evidence for a similar role of glutamate receptors in the nervous and immune systems. J Neurochem. (2005) 95:913–8. 10.1111/j.1471-4159.2005.03456.x 16271044

[280] Sarchielli P Di Filippo M Candeliere A Chiasserini D Mattioni A Tenaglia S. Expression of ionotropic glutamate receptor GLUR3 and effects of glutamate on MBP- and MOG-specific lymphocyte activation and chemotactic migration in multiple sclerosis patients. J Neuroimmunol. (2007) 188:146–58. 10.1016/j.jneuroim.2007.05.021 17628700

[281] Ganor Y Grinberg I Reis A Cooper I Goldstein RS Levite M. Human T-leukemia and T-lymphoma express glutamate receptor AMPA GluR3, and the neurotransmitter glutamate elevates the cancer-related matrix-metalloproteinases inducer CD147/EMMPRIN, MMP-9 secretion and engraftment of T-leukemia *in vivo*. Leuk Lymphoma. (2009) 50:985–97. 10.1080/10428190902878448 19391040

[282] Kaittanis C Andreou C Hieronymus H Mao N Foss CA Eiber M Prostate-specific membrane antigen cleavage of vitamin B9 stimulates oncogenic signaling through metabotropic glutamate receptors. J Exp Med. (2018) 215:159–75. 10.1084/jem.20171052 29141866 PMC5748857

[283] Banda M Speyer CL Semma SN Osuala KO Kounalakis N Torres Torres KE. Metabotropic glutamate receptor-1 contributes to progression in triple negative breast cancer. PLoS ONE. (2014) 9:e81126. 10.1371/journal.pone.0081126 24404125 PMC3880256

[284] Song Z He CD Liu J Sun C Lu P Li L. Blocking glutamate-mediated signalling inhibits human melanoma growth and migration. Exp Dermatol. (2012) 21:926–31. 10.1111/exd.12048 23171453

[285] Gelb T Pshenichkin S Hathaway HA Grajkowska E Dalley CB Wolfe BB. Atypical signaling of metabotropic glutamate receptor 1 in human melanoma cells. Biochem Pharmacol. (2015) 98:182–9. 10.1016/j.bcp.2015.08.091 26291396

[286] Gelb T Pshenichkin S Rodriguez OC Hathaway HA Grajkowska E DiRaddo JO. Metabotropic glutamate receptor 1 acts as a dependence receptor creating a requirement for glutamate to sustain the viability and growth of human melanomas. Oncogene. (2015) 34:2711–20. 10.1038/onc.2014.231 25065592 PMC5853109

[287] Renrick AN Dunbar ZT Shanker A. Update on the current revolution in cancer immunotherapy. Immunotherapy. (2019) 11:15–20. 10.2217/imt-2018-0135 30702010

[288] Entschladen F Palm D Niggemann B Zaenker KS. The cancer's nervous tooth: Considering the neuronal crosstalk within tumors. Semin Cancer Biol. (2008) 18:171–5. 10.1016/j.semcancer.2007.12.004 18249004

[289] Zanker S. The neuro-neoplastic synapse: does it exist? Prog Exp Tumor Res. (2007) 39:154–61. 10.1159/000100075 17314507

[290] Jiang SH Hu LP Wang X Li J Zhang ZG. Neurotransmitters: emerging targets in cancer. Oncogene. (2019) 39:503–15. 10.1038/s41388-019-1006-0 31527667

[291] Cole SW Nagaraja AS Lutgendorf SK Green PA Sood AK. Sympathetic nervous system regulation of the tumour microenvironment. Nat Rev Cancer. (2015) 15:563–72. 10.1038/nrc3978 26299593 PMC4828959

[292] Magnon C Hall SJ Lin J Xue X Gerber L Freedland SJ. Autonomic nerve development contributes to prostate cancer progression. Science. (2013) 341:1236361. 10.1126/science.1236361 23846904

[293] Cao L Liu X Lin EJ Wang C Choi EY Riban V. Environmental and genetic activation of a brain-adipocyte BDNF/leptin axis causes cancer remission and inhibition. Cell. (2010) 142:52–64. 10.1016/j.cell.2010.05.029 20603014 PMC3784009

[294] Hanoun M Zhang D Mizoguchi T Pinho S Pierce H Kunisaki Y. Acute myelogenous leukemia-induced sympathetic neuropathy promotes malignancy in an altered hematopoietic stem cell niche. Cell Stem Cell. (2014) 15:365–75. 10.1016/j.stem.2014.06.020 25017722 PMC4156919

[295] Saussez S Laumbacher B Chantrain G Rodriguez A Gu S Wank R. Towards neuroimmunotherapy for cancer: the neurotransmitters glutamate, dopamine and GnRH-II augment substantially the ability of T cells of few head and neck cancer patients to perform spontaneous migration, chemotactic migration and migration towards the autologous tumor, and also elevate markedly the expression of CD3zeta and CD3epsilon TCR-associated chains. J Neural Transm. (2014) 121:1007–27. 10.1007/s00702-014-1242-y 25030361

[296] Sun J Singh V Kajino-Sakamoto R Aballay A. Neuronal GPCR controls innate immunity by regulating noncanonical unfolded protein response genes. Science. (2011) 332:729–32. 10.1126/science.1203411 21474712 PMC3125668

[297] Mendez-Ferrer S Lucas D Battista M Frenette PS. Haematopoietic stem cell release is regulated by circadian oscillations. Nature. (2008) 452:442–7. 10.1038/nature06685 18256599

[298] Heidt T Sager HB Courties G Dutta P Iwamoto Y Zaltsman A. Chronic variable stress activates hematopoietic stem cells. Nat Med. (2014) 20:754–8. 10.1038/nm.3589 24952646 PMC4087061

